# METTL3/m^6^A/IFIT2 regulates proliferation, invasion and immunity in esophageal squamous cell carcinoma

**DOI:** 10.3389/fphar.2022.1002565

**Published:** 2022-10-20

**Authors:** Fangfang Ge, Zhenyu Li, Jiaru Hu, Youguang Pu, Fangfang Zhao, Lingsuo Kong

**Affiliations:** ^1^ Department of Anesthesiology, the First Affiliated Hospital of USTC, Division of Life Sciences and Medicine, University of Science and Technology of China, Hefei, China; ^2^ Department of Provincial Clinical College, Wannan Medical College, Wuhu, China; ^3^ Division of Life Sciences and Medicine, Department of the First Affiliated Hospital of USTC, University of Science and Technology of China, Hefei, China; ^4^ Division of Life Sciences and Medicine, Department of Cancer Epigenetics Program, The First Affiliated Hospital of USTC, University of Science and Technology of China, Hefei, China

**Keywords:** squamous cell carcinoma, N6-methyladenosine modification, METTL3, IFIT2, immune infiltration

## Abstract

Epigenetic regulation plays a critical role in the development, progression, and treatment of tumors. The most common chemical modification of mRNA, called m^6^A, is essential for controlling mRNA stability, splicing, and translation. Methyltransferase-like 3 (METTL3) is an important m6A methyltransferase. The mechanism of action of METTL3 in esophageal squamous cell carcinoma (ESCC) remains unclear. In this investigation, we sought to clarify the function and clinical importance of METTL3 in ESCC and investigate its underlying mechanisms. We discovered that METTL3 has a significant proliferative effect in ESCC cells by using lentiviral construction of stable cell lines overexpressing METTL3 (METTL3-OE) and knocking down METTL3 (sh-METTL3). To create a xenograft tumor model, we inoculated KYSE510 cells subcutaneously into BALB/c nude mice and discovered that sh-METTL3 inhibited the tumorigenicity of esophageal cancer KYSE510 cells in the nude mouse tumor model. MeRIP-seq and RNA-seq analysis revealed IFIT2 to be a METTL3 target gene. The findings revealed that METTL3 regulates IFIT2 and thus influences malignant biological behaviors such as proliferation, migration, and invasion of ESCC, as well as the immune microenvironment of tumors.

## Introduction

Esophageal cancer is the seventh most common malignant disease worldwide and the sixth leading cause of cancer-related mortality. Global cancer statistics for 2020 suggest 544,000 deaths and 604,000 new cases (Sung et al., 2021). One of the most aggressive tumors, squamous cell carcinoma, makes up 90% of metastatic esophageal cancers (Liu et al., 2017). Despite notable improvements in the treatment of lung, breast, and other cancers over the past ten years, the number of people with esophageal cancer has remained stable (Lagergren et al., 2017). Surgery, radiation therapy, and chemotherapy are all effective treatments for esophageal cancer, but their long-term prognosis is still poor (Wang et al., 2018). In an effort to improve the prognosis and overall survival rate of the disease, we are actively exploring the pathogenesis of esophageal cancer.

Genetic abnormalities are what initiates and fuels the development of cancer, and tumorigenesis is greatly influenced by epigenetic pathways (Dawson and Kouzarides, 2012). Epigenetic modifications can alter gene expression without changing the base sequence (Toh et al., 2017). RNA modification widely affects the structure, function and stability of RNA and has been a research hotspot in recent years (Barbieri and Kouzarides, 2020). mRNA is a crucial step in the process of interpreting genetic information since it is the main molecular connection between DNA and protein (Delaveau et al., 2016). One-third of all mammalian mRNAs have an average of 3-5 m^6^A modifications, which is the most common type of posttranscriptional modification in mRNA (Jiang et al., 2021). This modification has the potential to influence RNA stability, splicing, transport, and localization, and various diseases are influenced by its activity (Zhao et al., 2017). They are intently related to the regulation of current therapeutic approaches and may additionally supply new options for the fine cure of illness (Ma and Ji, 2020).

m^6^A modification is a dynamic and reversible process achieved by “writer” methylesterase, “eraser” demethylase and “reader” methyl recognition protein (Ping et al., 2014; Wang et al., 2016). METTL3, the major catalytic component of the methyltransferase complex, recognizes a conserved sequence of RNA 5′-RRACU-3' (R = A or G) (Dominissini et al., 2012). METTL3 is mainly involved in posttranscriptional regulation. Abnormal expression alters the fate of m^6^A transcripts (He and He, 2021). Loss of METTL3 results in a dramatic drop in m^6^A levels and effects on other molecules (Koh et al., 2019a). Researchers have found that METTL3 is highly expressed in lung adenocarcinomas, while METTL3-silenced cells proliferate and migrate much less than those that are not silenced (Lin et al., 2016). In contrast, METTL3 regulates tumor growth by cooperating with YTHDF2 to modify tumor-associated neutrophils (TANs) infiltration and performs a key tumor suppressor role in papillary thyroid carcinoma (He et al., 2021). It appears that METTL3 plays a dual role, which may be related to the tumor’s primary site of development, the cellular microenvironment, upstream and downstream regulatory elements, and resistance mechanisms.

The host immune system has an important influence on all aspects of tumor cell proliferation, epithelial mesenchymal transition, invasion, and metastasis (Liu et al., 2021). m^6^A modification also affects the tumor immune microenvironment (Ma et al., 2021). Inhibition of METTL3 enhances the response to immunotherapy in colorectal cancer and melanoma. Through T-cell activation, depletion, and infiltration mediated by PD-L1, METTL3 improves antitumor immunity in breast cancer both *in vitro* and *in vivo* (Wan et al., 2022). METTL3-mediated m^6^A modification has been shown to significantly improve the capacity of tumor-infiltrating myeloid cells to suppress the immune system and facilitate tumor immune evasion (Xiong et al., 2022). However, it is still unclear how METTL3 contributes to esophageal cancer and what its exact mechanism is. We also investigated whether METTL3’s role in esophageal cancer is immune-related.

In this study, we demonstrate that METTL3 regulates esophageal cancer proliferation, invasion, and immunity *via* the downstream target IFIT2.

## Materials and methods

### Esophageal cancer specimens and cell lines

Eleven samples from esophageal cancer patients who underwent surgery were obtained from the West Hospital of the First Affiliated Hospital of Chinese University of Science and Technology. The Biomedical Ethics Committee of the University of Science and Technology of China conducted every experiment for this study. The clinicopathological details of every participant who gave their written consent are listed in Supplementary Table S1. HEEC and KYSE150, KYSE510, KYSE30, KYSE140, KYSE410, and KYSE450 cell lines were obtained from the China Cell Resource Center (Shanghai, China). The cells were cultured in RPMI 1640 (Gibco) supplemented with 10% fetal bovine serum (PAN) and 1% penicillin‒streptomycin.

### RNA extraction and quantitative RT‒PCR analysis

Total RNA was extracted from tissues and cells using TRIzol reagent (Vazyme). A total of 1000 ng of total RNA was reverse transcribed to obtain cDNA using the HiScript®II 1st Strand cDNA Synthesis Kit (Vazyme). The relative mRNA expression was calculated using the 2^−ΔΔCt^ method. TaqMan probe quantitative PCR was used to locate the target gene’s expression, and ACTB was used as an internal control. All qPCR primer sequences are presented in Supplementary Table S2.

### Western blot

Lysis buffer was used to lyse esophageal tissue and cells. After boiling the proteins for 10 min in a metal bath, the proteins were denatured. SDS‒PAGE (Beyotime Biotechnology) was used to separate the samples, which were then transferred to PVDF membranes (Millipore). Then, the cells were blocked with 5% nonfat milk for an hour. The primary antibody (1:2000) was placed on the membrane overnight in a 4° refrigerator, and then the secondary antibody (1:5000) was applied to the membrane for 1 h at room temperature. The signal band was exposed to ECL luminescence solution (Thermo). Proteintech provided all the antibodies used in this article. Detailed information on full-length gels is provided in Supplementary Figures S7–S15.

### Plasmid and lentiviral transfection

The cells were seeded into 24-well plates, the cell density was increased to 30%, lentiviral infection (MOI = 3) was carried out, and puromycin was used to screen stable cell lines for overexpression and knockdown. Construction, sequencing, packaging, concentration, and purification were performed on the lentiviral plasmid containing the target gene. Virus titer determination was entrusted to Shanghai Hanheng Company. METTL3-OE (NM_019852.5) and IFIT2-OE (NM_001547.5). The IFIT2 gene was silenced using small interfering RNAs (siRNAs), all of which were produced by GenePharma. By using Lipofectamine 2000, plasmids containing the transgene and a packaging plasmid were cotransfected into KYSE510 and KYSE30 cells (Invitrogen, USA). The sh-RNA and siRNA sequences are listed in Supplementary Table S2.

### CCK-8 assay

The proliferation assay was carried out in a 96-well plate with 3000 cells per well. After 0, 24, 48 and 72 h, 10 μL of CCK-8 solution (Bimake) was added to every well and then incubated for 2 h. The absorbance (OD) of each well at 450 nm was detected by an enzyme labeling instrument, and the cell viability was calculated.

### Colony formation assay

In six-well plates, 300 or 400 cells were seeded per well and then cultured for two weeks in medium containing 10% FBS. After that, 4% paraformaldehyde was added for fixation for 15 min, followed by 0.1% crystal violet staining for 15 min. The staining solution was discarded, and the cells were air-dried and counted.

### Wound healing assays

Once the density reached 100% in twelve-well plates, the cells were scraped vertically from top to bottom with a 10 μL sterile pipette tip. Serum-free medium was added after washing 3 times with precooled PBS. Pictures were taken at 0 h and 24 h. Analysis of area measurements for wound healing experiments using ImageJ software.

### Transwell assay

Transwell chambers were covered with Matrigel (BD) for invasion assays, while those without Matrigel were used for migration assays. Differently treated cells (5 × 10^4^ for the migration assay and 1 × 10^5^ for the invasion assay) were loaded into 8 μm diameter 24-well transwells (Corning) and cultured without serum. In the lower compartment, 600 μL of medium containing 20% serum was added. Nonmigrating cells were scraped off after 24 h, and noninvasive cells were removed after 48 h. Migrating and invasive cells were fixed with 4% paraformaldehyde for 30 min. After staining with 1% crystal violet solution for 30 min, the cells in the upper chamber were wiped clean with a cotton swab, counted, and photographed.

### Flow cytometry

After being collected and resuspended in binding buffer, the cells underwent an incubation period of 5 min at room temperature with fluorescently labeled APC and PI (Annexin V-APC/PI apoptosis kit).

### Quantification of RNA m^6^A modification

To each well, 200 ng–300 ng of RNA extracted from cells, capture antibody, detection antibody, enhancer solution, chromogenic reagent solution, and stop solution was added and incubated in the dark for 5–15 min. The optical density value (OD) (450) of the standard was detected by a microplate reader at 450 nm and used to calculate the relative m^6^A content (m^6^A%) of the RNA to be tested. EpiQuik M^6^A RNA methylation quantitative kit (P9005; Epigentek).

### RNA-sequencing, RNA-seq

The RNA library construction kit was used to build the library after the RNA had been quantified, and all operations were carried out in accordance with the instructions. Second-generation sequencing was carried out by Ribo after the library was checked, double end sequenced, and used in a sequencer.

### Model of xenotransplantation

ESCC tumor xenograft model establishment in 4-week-old male nude mice. Mice were subcutaneously injected with 1 × 10^7^ cells resuspended in 100 μL PBS containing Matrigel (1:1). Every four days, the tumor volume was measured with calipers. The volume is calculated as 1/2×length×width squared. One month after injection, the mice were euthanized. The weight and images of subcutaneous tumors were taken. The mouse tumors were embedded and fixed before being stained with HE on dewaxed sections. IHC staining of Ki67 revealed the proliferation index. TUNEL was used to detect apoptotic cells. All animal research procedures were carried out under a program approved by the Animal Laboratory Center of University of Science and Technology of China.

### Immunohistochemistry

Dewaxed and hydrated paraffin sections were incubated with the primary antibodies Ki67 (1:200), METTL3 (1:500) and IFIT2 (1:500) overnight at 4°C before being incubated with the secondary antibody for two hours at room temperature. The antigen was then repaired and titrated with a blocking endogenous peroxidase blocker.

For 7 pairs of cancer and paracancerous tissue samples, the H-Score method was used to analyze them by ImageJ’s IHC Profiler software (Varghese et al., 2014). The result obtained was scored as 0 (negative), 1+ (low positive), 2+ (positive) and 3+ (high positive). For IHC staining of METTL3, an IHC score ≥2 was defined as high METTL3 expression, and an IHC score <2 was defined as low METTL3 expression. Ki67 was detected in animal specimens, and the positive area of Ki67 was counted by ImageJ.

### Bioinformatics analysis

The Gene Expression Profiling Interactive Analysis (GEPIA) database was used to analyze the total expression level of METTL3 in ESCC and normal esophageal epithelial samples. The TCGA dataset was used to download the TRNA-sequencing expression profiles for ESCC along with the associated clinical data. Based on the prognostic data of esophageal cancer patients and METTL3 and IFIT2 expression data, a prognostic model was constructed using the LASSO Cox regression model (R package “glmnet”). The risk score was calculated by normalizing the TCGA expression data (R package “scale” function) with the following formula: Risk Score = (X: regression coefficient; Y: gene expression level), and the ROC prognostic assessment curve was constructed based on the risk score.

ROC curve analysis by muti_cox. R package and pROC package. The pathways that were enriched by GSEA. TIMER is a convenient method for analyzing immune infiltrates in TCGA tumors. Multigene correlation was visualized using the R programming language’s pheatmap package.

### Statistical analysis

The experimental results were analyzed using GraphPad Prism 9.0 statistical software. The data are at least the average of three experimental data points, and the *t* test was used to compare groups and analyze differences between them (*p < 0.05, **p < 0.01, ***p < 0.001, ****p < 0.0001).

## Results

### METTL3 is upregulated in esophageal squamous cell carcinoma

**FIGURE 1:**
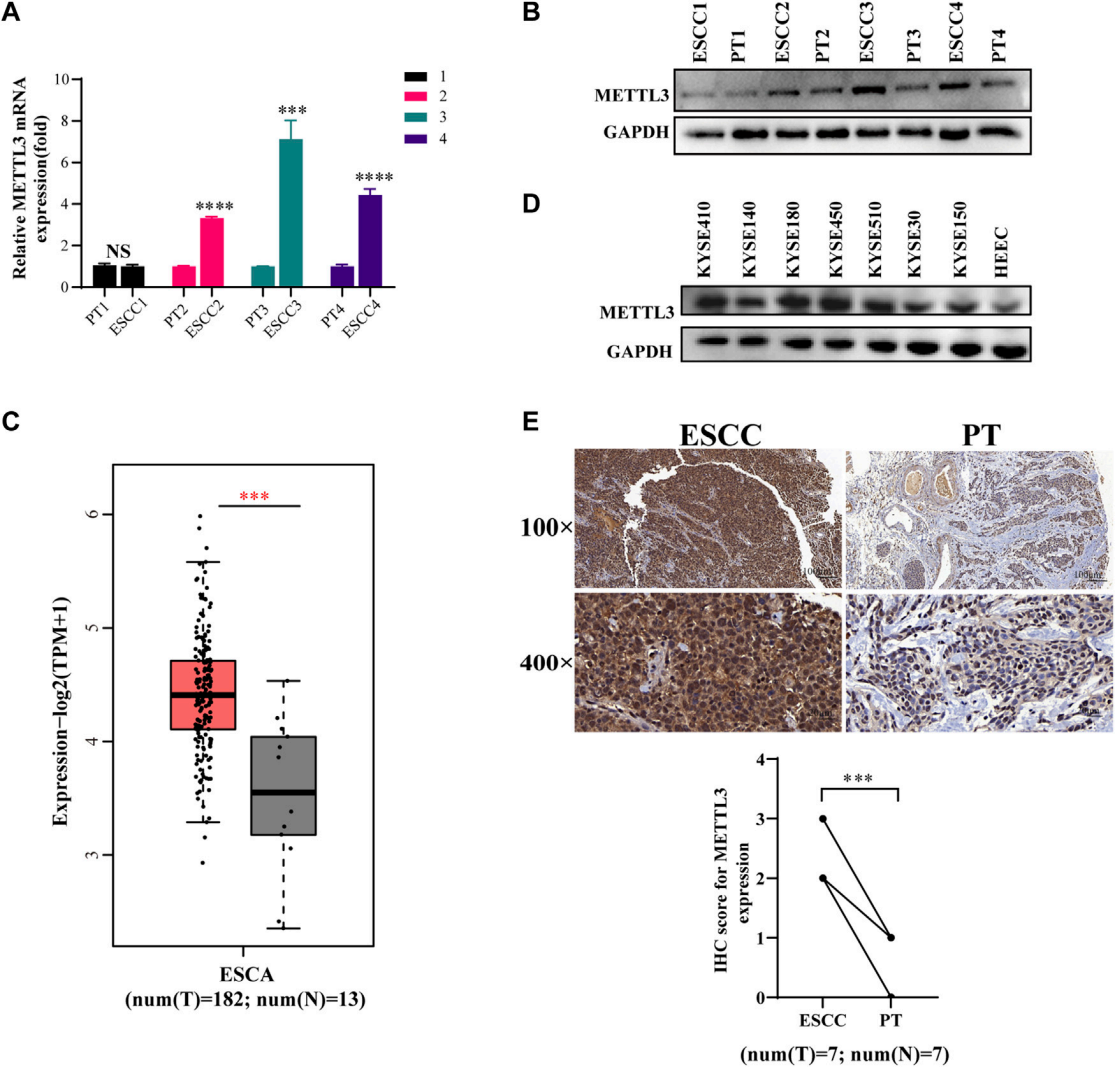
METTL3 is upregulated in esophageal squamous cell carcinoma **(A,B)**. qPCR and WB were used to measure METTL3 expression in 4 pairs of ESCC tissues and corresponding adjacent tissues. **(C)**. METTL3 expression levels in the TCGA database. **(D)**. METTL3 was expressed in seven ESCC cell lines and HEEC cells. **(E)**. Representative image of immunohistochemical staining by METTL3 in 100-fold (scaled bar = 100 μm) and 400-fold (scaled bar = 20 μm) magnified ESCC tissues and paired normal tissues in human samples (above). Immunohistochemical expression of METTL3 in ESCC tumor tissue and paired paracancerous tissues (PT) was quantitatively analyzed using ImageJ Profiler software (*n* = 7). n.s, no statistical significance, ***p < 0.001, ****p < 0.0001.

We gathered 11 pairs of clinical samples from esophageal cancer and paracancerous tissue. Following the extraction of RNA and protein from 4 pairs, it was discovered that the levels of METTL3 mRNA and protein expression in tumor tissues were higher than those in adjacent tissues (Figures 1A,B). This is in line with the TCGA database’s findings that esophageal cancer tumor tissues exhibit high levels of METTL3 expression (Figure 1C). Seven esophageal cancer cell lines expressed METLL3 at levels higher than those of human esophageal epithelial cells (HEECs) (Figure 1D). After that, immunohistochemistry was used to examine seven pairs of clinical specimens that had been fixed, dehydrated, and paraffin-embedded, and the results revealed that METTL3 expression was higher than that of adjacent tumors (Figure 1E). Collectively, these results clearly demonstrated that METTL3 is highly expressed in esophageal cancer patients.

### High METTL3 expression is associated with ESCC proliferation, apoptosis, migration and invasion

**FIGURE 2:**
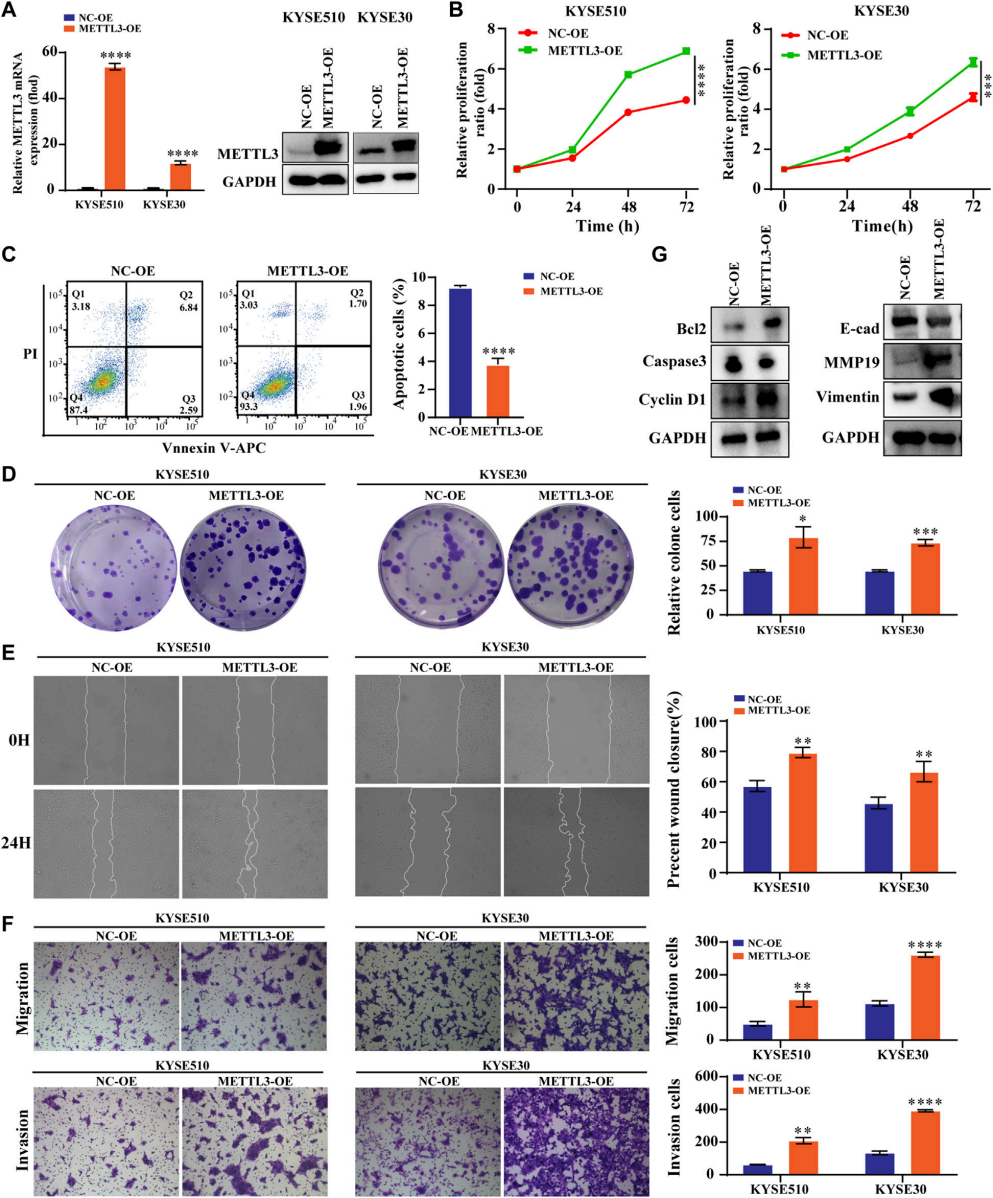
High METTL3 expression is associated with proliferation, apoptosis, migration and invasion in ESCC cells **(A)**. METTL3 mRNA and protein levels were drastically elevated following lentiviral transfection of KYSE510 and KYSE30 cells. **(B)**. The CCK-8 assay assessed the proliferation ability of METTL3-overexpressing KYSE510 and KYSE30 cells. **(C)**. Flow cytometric analysis was conducted to assess apoptosis in KYSE510 METTL3-overexpressing cells. **(D)**. The clonality of KYSE510 and KYSE30 cells was measured by the plate clone. **(E)**. The wound scratch test and **(F)**. Transwell migration and invasion assays were conducted to examine the migratory and invasion capacities of KYSE510 and KYSE30 cells overexpressing METTL3. **(G)**. Expression of E-cadherin, MMP19 and Vimentin proteins was detected *via* western transfer in METTL3-overexpressing cells. The protein levels of Bcl2, Caspase 3 and Cyclin D1 were detected by western blotting in METTL3-overexpressing cells. *p < 0.05, **p < 0.01, ***p < 0.001, ****p < 0.0001.

We constructed stable cells using lentiviral METTL3 expression constructs. As shown, the results indicated that not only the METTL3 expression of this protein but also the expression at the mRNA level was increased (Figure 2A). The results from CCK-8 and colony experiments revealed that overexpressing METTL3 significantly increased the ability of KYSE510 and KYSE30 cells to proliferate. Likewise, flow cytometry measurements were employed to evaluate the apoptotic capacity of cells. These results confirmed that METTL3 overexpression notably decreased the percentage of apoptotic cells (Figure 2B–D). Wound-healing assays showed that METTL3-overexpressing cells had a markedly increased wound closure area at 24 h (Figure 2E). To similarly reflect the effect of METTL3 on cell invasion, transwell invasion and migration assays were performed. The results implied that METTL3 overexpression promoted cell migration and invasion (Figure 2F). In the detection of associated apoptosis proteins, BCL2 and cyclin D1 protein levels were upregulated in the METTL3-OE group. At the same time, overexpressing METTL3 inhibited the expression of Caspase3. Moreover, we noted that the expression of EMT-related proteins varied, with MMP19 and Vimentin showing increased expression, while E-cadherin showed diminished expression (Figure 2G).

### Low METTL3 expression is associated with proliferation, apoptosis, migration and invasion in ESCC cells

**FIGURE 3:**
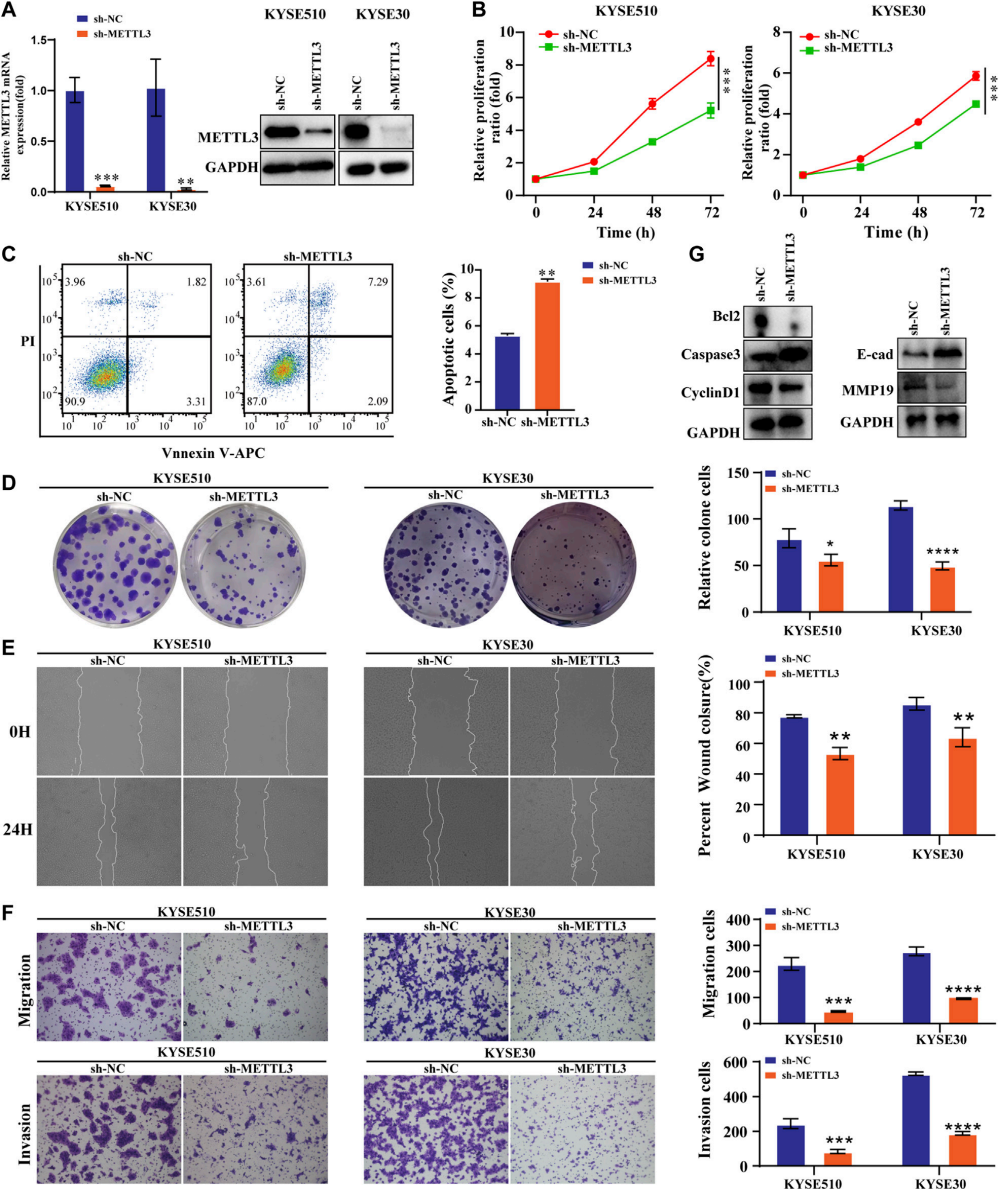
Low METTL3 expression is associated with proliferation, apoptosis, migration and invasion in ESCC cells **(A)**. The mRNA and protein levels of METTL3 were drastically decreased following lentiviral transfection of KYSE510 and KYSE30 cells. **(B)**. The CCK-8 assay assessed the proliferation ability of METTL3-knockdown cells. **(C)**. Flow cytometric analysis was conducted to assess apoptosis in KYSE510 METTL3-knockdown cells. **(D)**. The clonality of KYSE510 and KYSE30 cells was measured by the plate clone. **(E)**. The wound scratch test and **(F)**. Transwell migration and invasion assays were conducted to examine the migratory and invasion capacities of METTL3 knockdown cells. **(G)**. Expression of E-cadherin and MMP19 proteins was detected *via* western transfer in METTL3-knockdown cells. The protein levels of Bcl2, Caspase 3 and Cyclin D1 were detected by western blotting in METTL3-knockdown cells. *p < 0.05, **p < 0.01, ***p < 0.001, ****p < 0.0001.

We performed the assay utilizing shRNA lenticular METTL3 knockdown. The results confirmed that the expression of this protein at the mRNA level as well as at METTL3 was reduced (Figure 3A). The viability and proliferation of cells were markedly reduced by METTL3 knockdown, as tested through CCK8 and colony formation assays. In contrast, sh-METTL3 significantly promoted apoptosis (Figures 3B–D). We further explored the invasive and migratory abilities of the cells. The scratch healing ability of the sh-METTL3 group was slowed (Figure 3E). The low expression of METTL3 in the invasion assay reduced the number of invading cells, which was consistent with the results of the migration assay (Figure 3F). The apoptosis and expression of EMT-related proteins after METTL3 knockdown were opposite to those after METTL3 overexpression (Figure 3G).

### MeRIP-seq analysis of the effect of overexpression on the methylation of target genes

**FIGURE 4:**
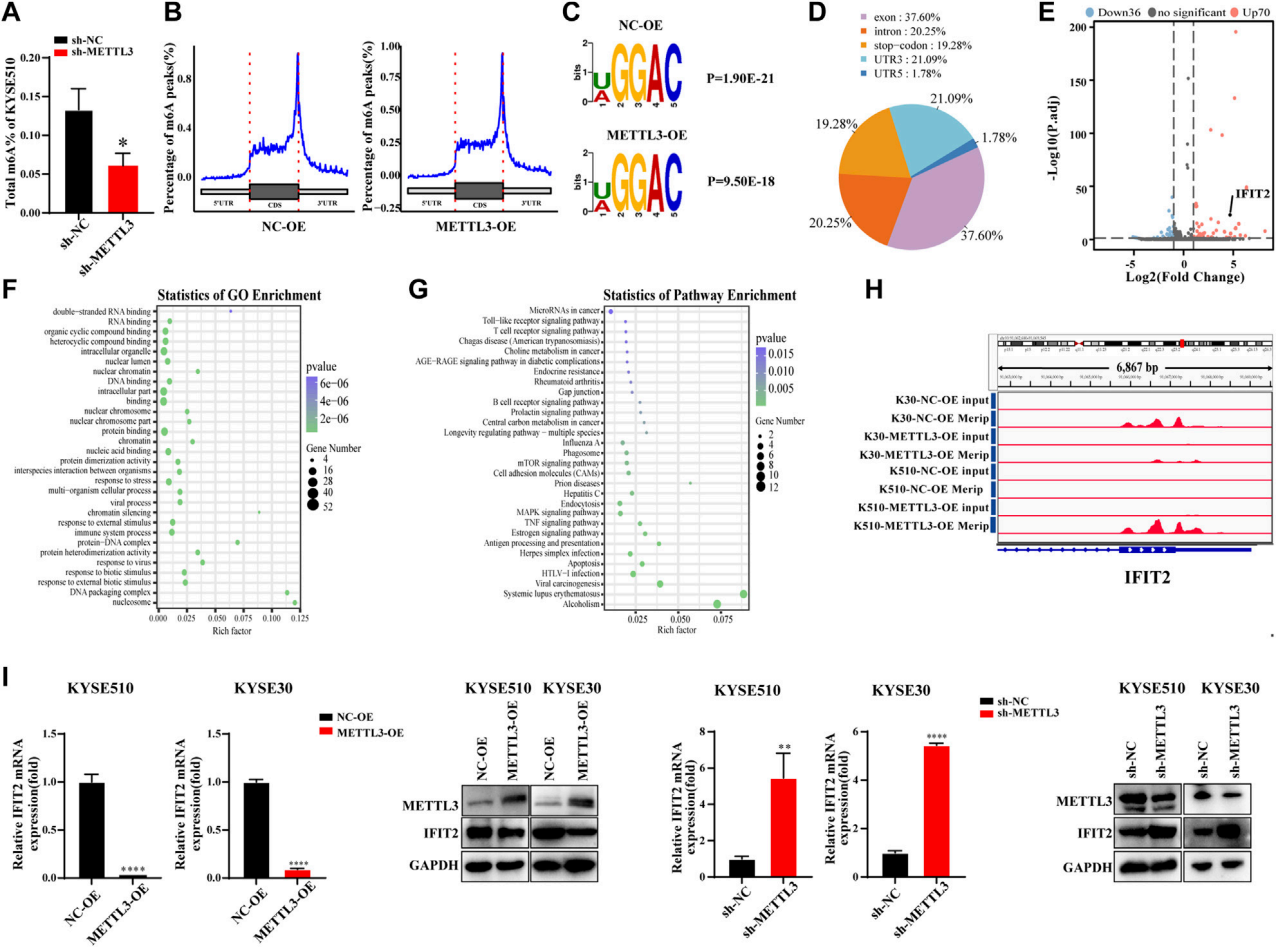
MeRIP-seq analysis of the effect of overexpression on the methylation of target genes **(A)**. The m^6^A RNA methylation assay revealed the m^6^A content in KYSE510 cells with METTL3 **(B)**. Peak distribution in CDS, 5′UTR and 3′UTR regions (The abscissa is the CDS, 5′UTR, and 3′UTR regions, and the ordinate is the distribution ratio of the peaks corresponding to the positions of each region). **(C)**. The predominant consensus motif GGAC was detected in m6A-seq. **(D)**. Statistical results of the regional distribution of the difference peak. **(E)**. The volcano plot shows the distribution of differential genes. **(F)**. The results of GO biological process enrichment. **(G)**. The results of KEGG pathways analysis. **(H)**. m^6^A peaks were enriched from m^6^A RIP-seq data. **(I)**. Effects of METTL3 overexpression or knockdown on IFIT2 mRNA and protein expression in KYSE510 and KYSE30 cells. *p < 0.05, **p < 0.01, ****p < 0.0001.

We detected the m^6^A contents of the total mRNA in METTL3-silenced KYSE510 cells. As expected, METTL3 silencing dramatically decreased the m^6^A content in KYSE510 cells (Figure 4A). Taking the sample as the unit to annotate the peaks of the sample and counting and annotating the annotation results, the results show that the m^6^A peaks are abundant in coding sequences (CDS), especially near stop codons in the 3′UTR of mRNA (Figure 4B). The RMBase database determined that the consensus motif for the genes with m^6^A modification is U/AGGAC (*p* = 2.3e-022), which is the common feature among the genes with m^6^A methylation (Figure 4C). The main distribution area of the difference peak was in the exon (37.6%), intron (20.25%), 3′UTR (21.09%) and stop codon (19.28%). Only a small portion (1.78%) was distributed in the 5′UTR (Figure 4D). The significantly differentially expressed genes between samples can be further selected by the fold difference and significance level. Notably, 70 transcripts were upregulated, and 36 transcripts were downregulated in the RNA-seq and MeRIP-seq data (Figure 4E and Supplementary Figure S1). We took the intersection of the downregulated peak after overexpression of METTL3 and the upregulated peak after interference with METTL3 and then enriched the GO and KEGG functions of the intersected genes. GO analyses showed that immune system processes were significantly enriched. KECG has been shown to be associated with immune signaling pathways, including the Toll-like, T-cell and B-cell receptor signaling pathways. Moreover, genes were also significantly enriched in apoptosis (Figures 4F,G and Supplementary Figures S2, S3). Among these genes, we chose and verified the gene that was most strongly associated using qRT‒PCR and WB. We thus detected the m^6^A abundance on IFIT2 mRNA transcripts in KYSE30 and KYSE150 cells by m^6^A-seq, and the results showed that m^6^A methylation was enriched in the exon and 3′UTR regions of IFIT2 with a clustered distribution (Figure 4H). We then characterized the expression relationship of IFIT2 with METTL3 in esophageal cancer cells. Notably, IFIT2 mRNA and protein expression were both significantly downregulated by METTL3 overexpression in KYSE510 cells (Figure 4I), whereas IFIT2 mRNA and protein expression were upregulated by METTL3 knockdown in KYSE510 cells (Figure 4I).

### IFIT2 overexpression inhibited ESCC cell proliferation and invasion

**FIGURE 5:**
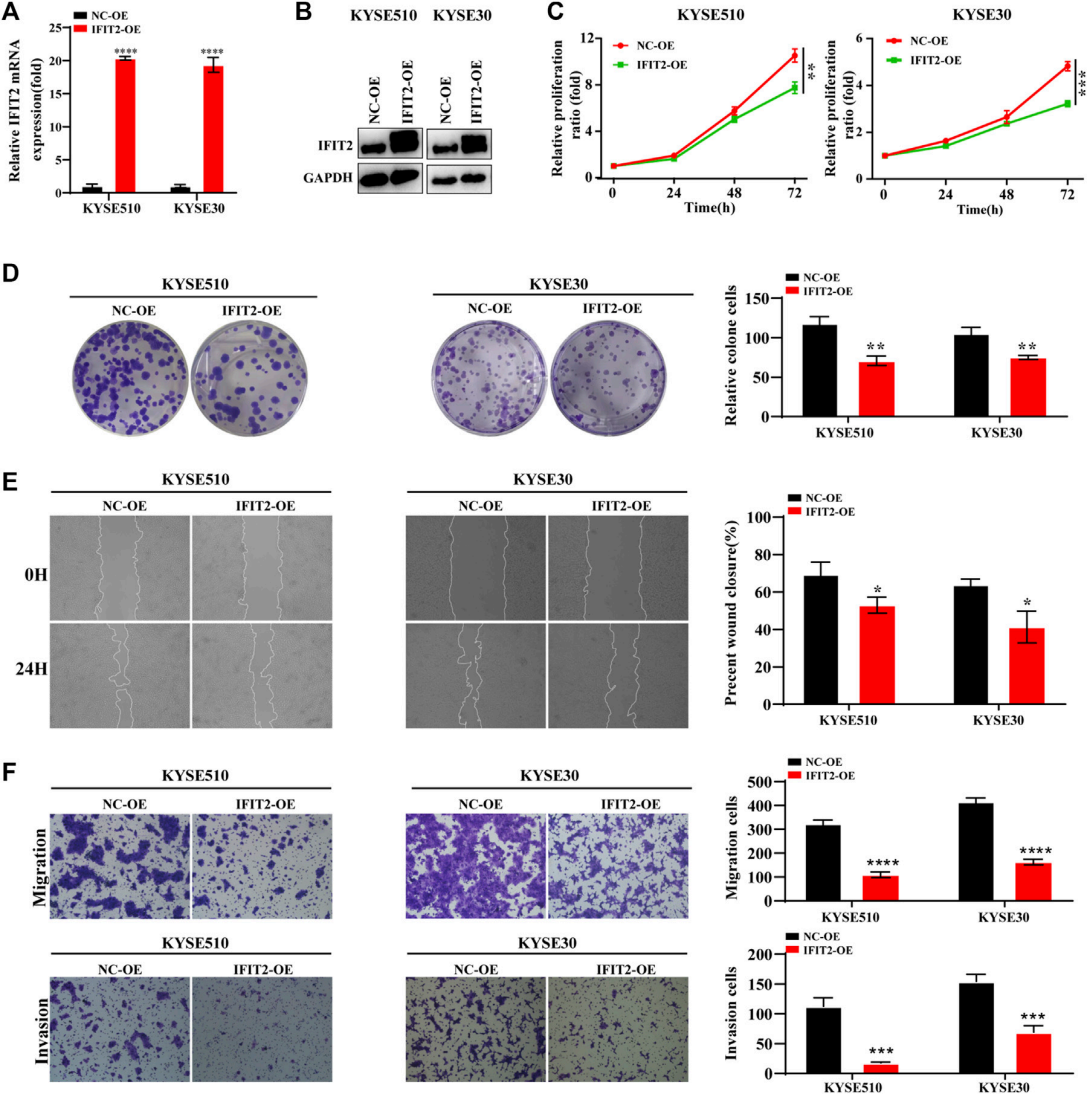
IFIT2 overexpression inhibited ESCC cell proliferation and invasion **(A, B)**. To evaluate the effectiveness of the IFIT2 overexpression lentivirus, qRT‒PCR was used to detect mRNA levels, and western blot analyses were used to detect protein levels. **(C)**. A CCK-8 assay was used to evaluate the proliferation ability of KYSE510 and KYSE30 cells after IFIT2 overexpression. **(D)**. Cell proliferation capabilities were identified through colony formation tests in transfected KYSE510 and KYSE30 cells. **(E)**. The wound healing assay reduced the KYSE510 and KYSE30 cell ability to migrate. **(F)**. Transwell assays were used to analyze transfected cell migration and invasion. *p < 0.05, **p < 0.01, ***p < 0.001, ****p < 0.0001.

The IFIT2 transfection efficiency was confirmed to be increased at both the mRNA and protein levels (Figure 5A, B). Next, we verified the function of downstream target genes of IFIT2. The results of CCK-8 and cloning experiments showed that IFIT2 overexpression attenuated the proliferation of esophageal cancer cells (Figures 5C,D). IFIT2 overexpression prevented cell migration, according to scratch experiments (Figure 5E). It was determined whether IFIT2 in cells affected cell invasion and migration using a transwell assay. These cells displayed less cell migration and invasion (Figure 5F).

### Effect of IFIT2 siRNA on ESCC cell proliferation, migration, and invasion

**FIGURE 6:**
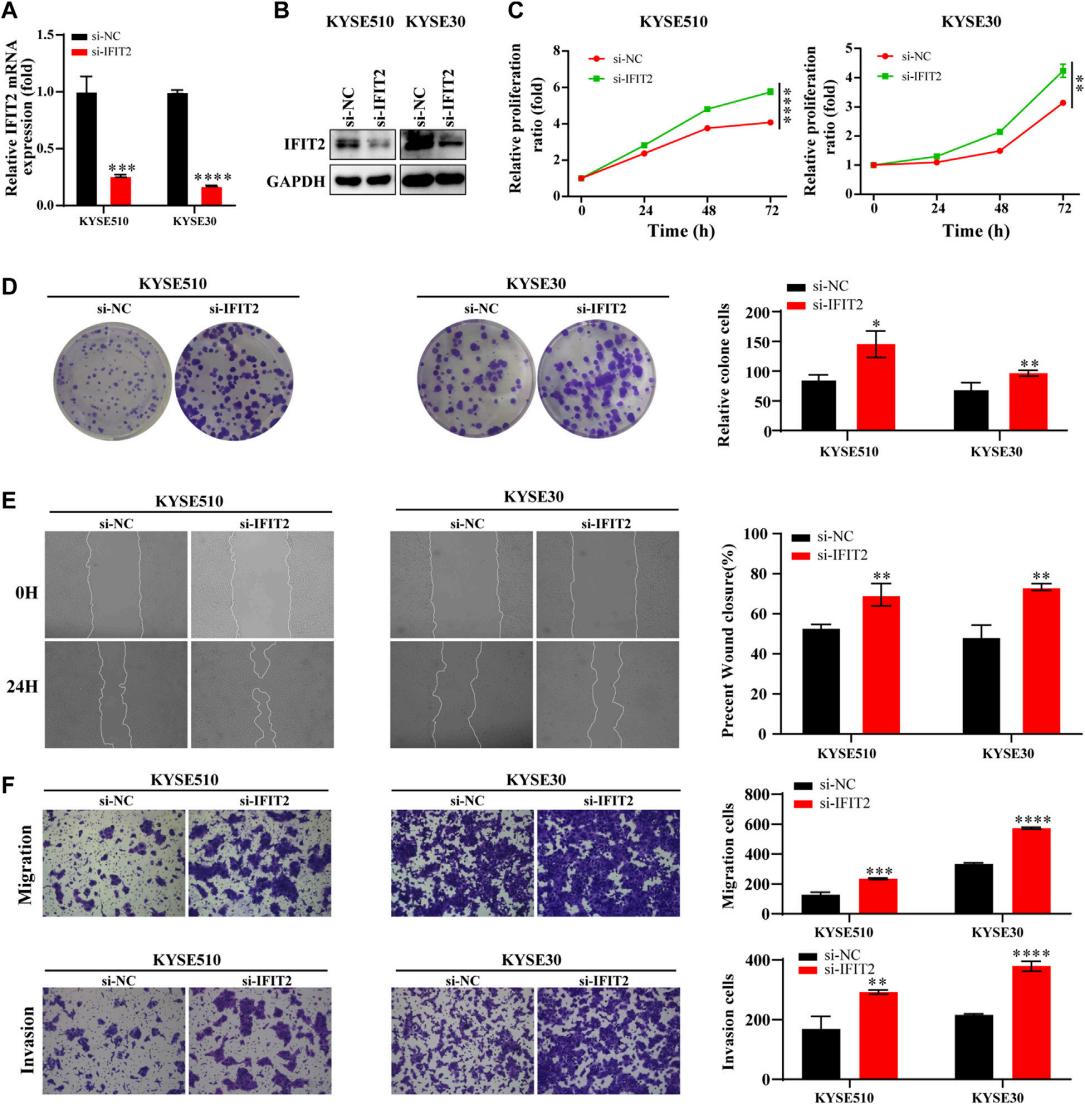
Effect of IFIT2 siRNA on ESCC cell proliferation, migration, and invasion **(A, B)**. To evaluate the effectiveness of si-IFIT2, qRT‒PCR was used to detect mRNA levels, and western blot analyses were used to detect protein levels. **(C)**. A CCK-8 assay was used to evaluate the proliferation ability of KYSE510 and KYSE30 cells after si-IFIT2. **(D)**. Cell proliferation capabilities were identified through colony formation tests in transfected KYSE510 and KYSE30 cells. **(E)**. The healing experience reduced the KYSE510 and KYSE30 cell ability to migrate. **(F)**. Transwell assays were used to analyze transfected cell migration and invasion. *p < 0.05, **p < 0.01, ***p < 0.001, ****p < 0.0001.

The transfection efficiency of si-IFIT2 was confirmed at both the protein and mRNA levels (Figures 6A,B). siRNA-IFIT2-transfected KYSE510 and KYSE30 cells significantly enhanced cell proliferation, as shown by CCK8 and colony formation assays (Figures 6C,D). In comparison to control and untransfected siRNA, the scratch wound healing and migration assays showed that siRNA transfection significantly accelerated cell migration and facilitated wound healing and increased the number of cell invasions (Figures 6E,F).

### METTL3 reverses the biological effects of IFIT2 on ESCC proliferation, invasion, and metastasis

**FIGURE 7:**
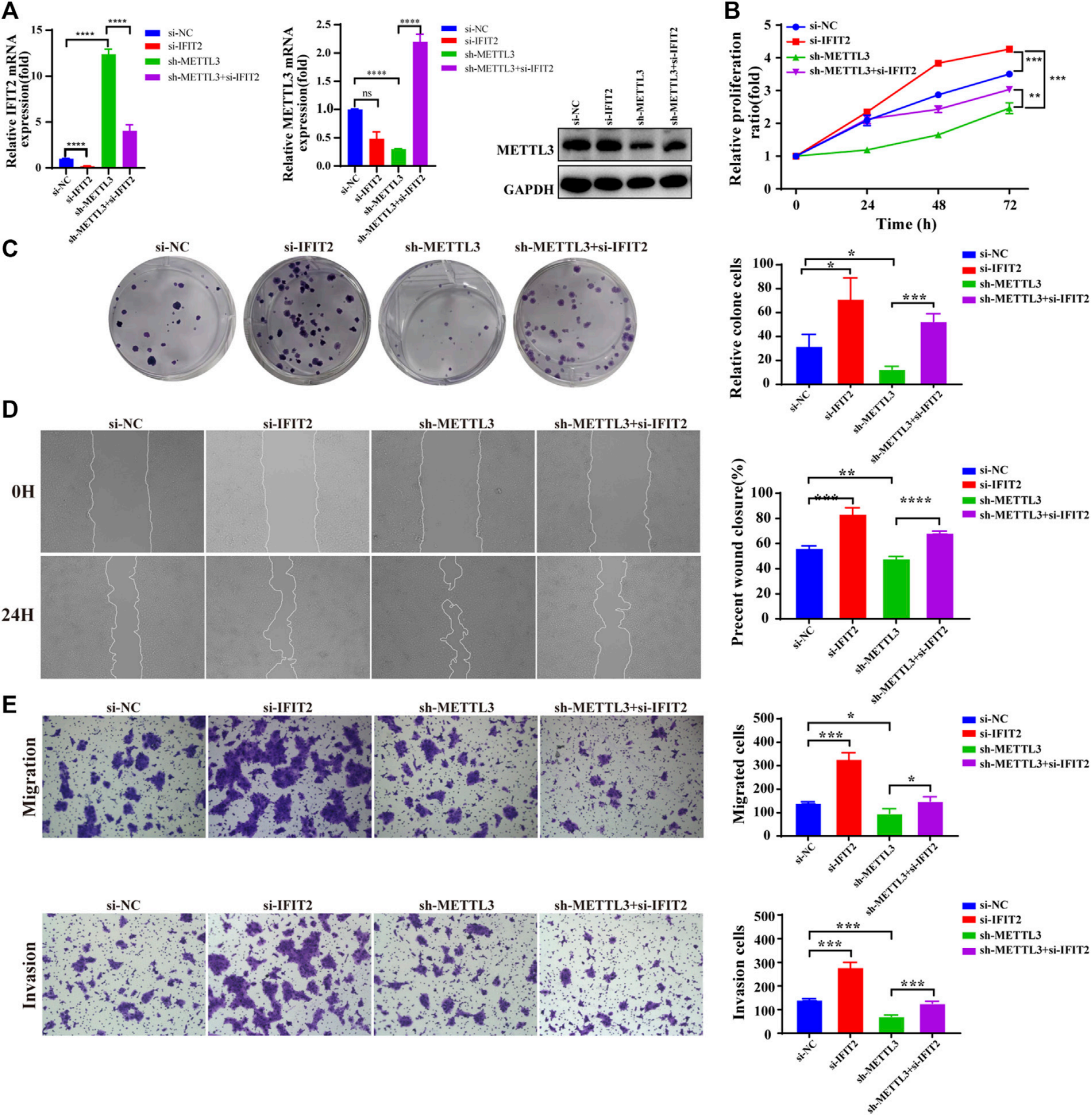
METTL3 reverses the biological effects of IFIT2 with respect to ESCC proliferation, invasion and metastasis **(A)**. Effects of siRNA-IFIT2 on METTL3 mRNA and protein expression following METTL3 knockdown. **(B,C)**. In the CCK-8 and colony formation assays, the inhibition of KYSE150 cell proliferation caused by poorly regulated METTL3 was restored by increased regulation of IFIT2 expression. **(D)**. The wound healing of cells demonstrated that upregulating IFIT2 restored the inhibition of METTL3-induced cell migration. **(E)**. The transwell migration and invasion tests were successful in rescuing the inhibition of migration and invasion capacity induced by sh-METTL3. n.s, no statistical significance, *p < 0.05, **p < 0.01, ***p < 0.001, ****p < 0.0001.

To further investigate the relationship between METTL3 and IFIT2 in the tumorigenesis of esophageal cancer, we knocked down IFIT2 in KYSE150 cells, accompanied by METTL3 knockdown. In siRNA-IFIT2-transfected cells, IFIT2 mRNA levels were decreased 6.5-fold, whereas IFIT2 expression was increased 12-fold in METTL3-silenced KYSE150 cells. IFIT2 expression in METLL3-silenced KYSE150 cells transfected with the si-IFIT2 vector (sh-METTL3+si-IFIT2) increased approximately 3-fold. Meanwhile, we confirmed that si-IFIT2 partially rescued METTL3 mRNA and protein upregulation in sh-METTL3 cells (Figure 7A). In fact, the reduction in KYSE510 cell proliferation following METTL3 knockdown was rescued by ectopic expression of IFIT2, as shown by CCK-8 and colony formation assays (Figures 7B,C). The expression of IFIT2 during wound healing partially rescued sh-METTL3 cells in the wound healing area (Figure 7D). *In vitro* cell migration and invasion assays, si-IFIT2 effectively restored the mechanical properties of METTL3 knockdown cells (Figure 7E).

### *In vivo* experimental validations in mice

**FIGURE 8:**
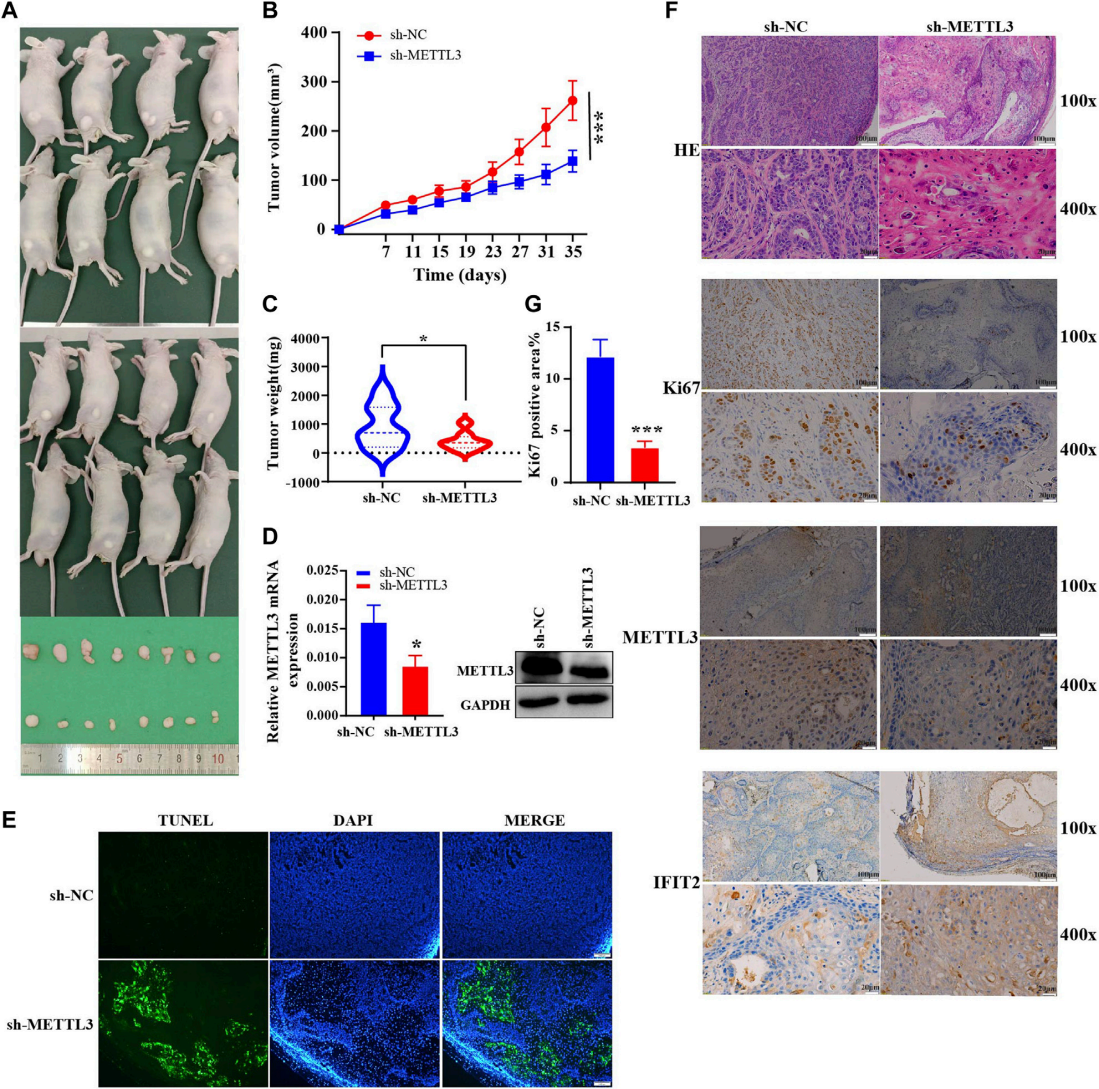
*In vivo* experimental validations in mice **(A)**. An analysis of the shape of transplanted tumors, changes in body weight of nude mice **(B)** and growth curves of tumor volume for each group **(C)** (*n* = 8). **(D)**. Expression of mouse tumor protein and mRNA. **(E)**. TUNEL staining of the paraffin sections of the xenograft tumors. Evaluation of tumor cell apoptosis by TUNEL staining (scale bar = 100 μm). **(F)**. Ki67 expression and HE coloration in mouse tumor tissue. Immunohistochemical recovery of the expression of METTL3 and IFIT2 (Scale bars are indicated in the figure). **(G)**. The ratio of KI67-positive area in the sh-NC and sh-METTL3 groups was analyzed using ImageJ software. *p < 0.05, ***p < 0.001.

We investigated the effect of METTL3 on tumorigenicity *in vivo* using a xenograft model of sh-METTL3 KYSE510 cells. Mice were inoculated subcutaneously with KYSE510 sh-NC and sh-METTL3 cells, and tumor size were measured every 4 days. Thirty-five days after tumor cell injection, subcutaneous tumor tissue was dissected, and tumor weight and volume were measured (Figure 8A). The tumor growth rate in the experimental group was substantially inhibited, and the tumor volume was significantly reduced (Figures 8B,C). By extracting RNA and proteins from mouse tumors, METTL3 was effectively silenced in the sh-METTL3 group (Figure 8D). After TUNEL staining, the range of TUNEL-positive cells improved in sh-METTL3 tumor tissue sections, consistent with the tumor size results (Figure 8E). Under the light microscope with HE staining in the transplanted tumor tissue, the nuclear volume of the tumor cells increased, as did the nuclear-cytoplasmic ratio. Meanwhile, immunohistochemistry also showed that METTL3 was successfully knocked out. METTL3 and IFIT2 were negatively correlated with immunohistochemistry (Figure 8F). In addition, the proliferation marker Ki67 was noticeably lower in sh-METTL3 cells than in sh-NC cells, according to IHC staining of the resected tumor tissue. ImageJ software statistics showed that the positive area of Ki67 in the sh-NC group was higher than that in the sh-METTL3 group (Figure 8G).

### METTL3 and IFIT2 are associated with prognostic immune infiltration

**FIGURE 9:**
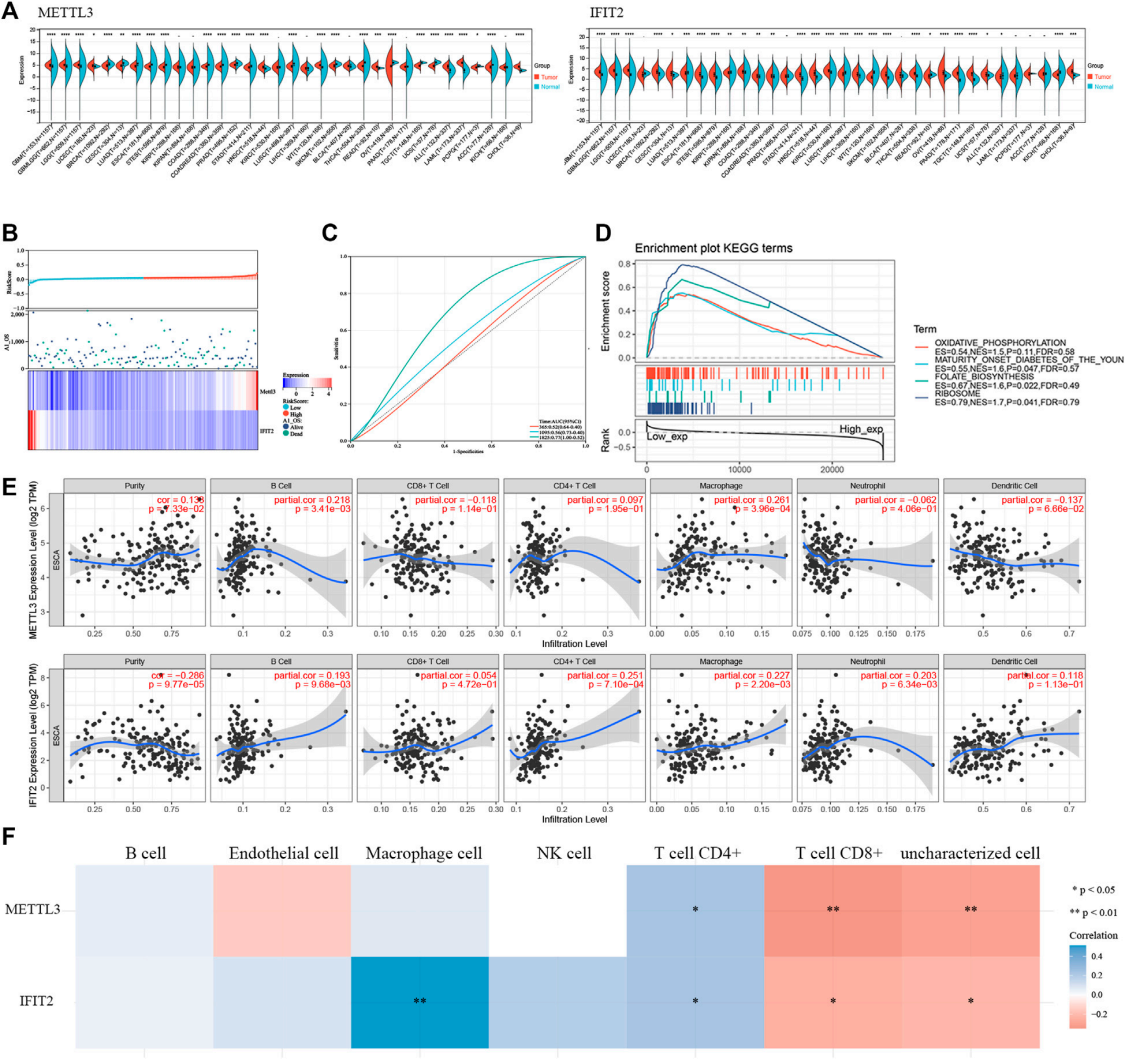
METTL3 and IFIT2 are associated with prognostic immune infiltration **(A)**. Pancancer analysis of METTL3 and IFIT2 expression across cancers from TCGA. **(B)**. The expression profiles that correspond to the distributions of prognostic signature-based risk scores. Low-risk patients are represented by green dots, while high-risk patients are represented by red dots. **(C)**. The ROC curve for evaluating the prediction efficiency of the prognostic signature. **(D)**. Correlation of IFIT2 gene expression and pathway enrichment in TCGA esophageal cancer samples by GSEA enrichment analysis. **(E)**. Correlation of IFIT2 and METTL3 expression levels with immune infiltration of B cells, CD4 T cells, CD8 T cells, neutrophils, macrophages, and dendritic cells in ESCC. **(F)**. A heatmap of the correlation between METTL3, IFIT2 and immune score.

When we used TCGA pancancer data to analyze the expression levels of the model’s key genes, METTL3 and IFIT2 were observed to have extensively different expression levels in regular and tumor tissues (Figure 9A). We developed a prognostic model using the genes METLL3 and IFIT2. According to the median risk score, ESCC cancer patients were divided into two groups: low-risk and high-risk (Figure 9B). Kaplan‒Meier (K-M) analysis was utilized to explore the prognostic value of scores (Supplementary Figure S4). ROC curve analysis showed that the risk model constructed by the expression and prognosis of IFIT2 and METTL3 in esophageal cancer patients was closely related to the prognosis survival of more than 3 years (AUC = 0.77) (Figure 9C). In TCGA esophageal cancer samples, IFIT2 gene expression and pathway enrichment were correlated by GSEA enrichment analysis. The findings demonstrate that IFIT2 expression is linked to the suppression of the oxidative phosphorylation pathway in tissues from esophageal cancer (Figure 9D). The tumor immune microenvironment is associated with the prognostic survival of patients. Therefore, we investigated the correlation of METTL3 and IFIT2 expression with immune cells in esophageal cancer. The findings of the TIMER database showed that the expression of METTL3 was appreciably correlated with the diploma of infiltration of B cells (*p* = 3.41e-03) and macrophages (*p* = 3.96e-04). IFIT2 was significantly inversely correlated with tumor purity (*p* = 9.77e-05), B cells (*p* = 9.68e-03), CD4^+^ T cells (*p* = 7.10e-04), macrophages (*p* = 2.20e-03) and neutrophils (*p* = 6.34e-03) (Figure 9E). We performed multigene correlation analysis using the R package pheatmap, and METTL3 and IFIT2 showed a negative correlation. METLL3 was significantly correlated with CD8^+^ T cells and uncharacterized cells (*p* < 0.01). IFIT2 was correlated with CD8^+^ T cells and uncharacterized cells (*p* < 0.05) (Figure 9F).

## Discussion

Studies have shown that proteins related to m^6^A modification are dysregulated and carcinogenic in esophageal cancer. Wang W found that METTL3 expression was significantly elevated in esophageal squamous cell carcinoma and was associated with poor patient prognosis (Han et al., 2021; Wang et al., 2021). FTO demethylase overexpression enhances esophageal cancer cell proliferation and tumor development (Cui et al., 2021). In a prior study, our team discovered that ESCC had significantly higher FTO levels, which in conjunction with ERBB2 controlled ESCC tumorigenesis and metastasis (Zhao et al., 2022). Li J showed that in the pathophysiology of ESCC, ALKBH5 acts as a tumor suppressor (Li et al., 2021). Due to advances in biological technologies such as high-throughput sequencing and the discovery of abnormal expression of METTL3, ALKBH5, and FTO, the role of m^6^A methylation in ESCC has gradually been revealed (Zhang et al., 2021). The insidious onset of ESCC is primarily responsible for the patients’ poor prognosis, and by the time of diagnosis, the majority had advanced to the middle and late stages of lymphatic metastasis. As a result, to find reliable prognostic biomarkers and therapeutic targets, it is imperative to research the molecular pathogenesis of esophageal cancer.

METTL3 is the most important methylase of m^6^A. In recent years, it has become clear that METTL3 is aberrantly expressed in gastric (Yue et al., 2019), breast (Pan et al., 2021), prostate (Chen et al., 2021) and non-small cell lung cancers (Xue et al., 2021), which raises the possibility that METTL3 could be a useful diagnostic marker and therapeutic target (Zeng et al., 2020). Our study shows that METTL3, a protein highly expressed in esophageal cancer tissues, can promote the proliferation, invasion and metastasis of esophageal cancer both *in vitro* and *in vivo*. These results are consistent with previously reported results. Although the trend of m^6^A modification levels is constant, it may have different effects by controlling different target genes, and the impact on tumor prognosis may be different (Li et al., 2019). In this study, we mainly found that METTL3 affects the occurrence and development of ESCC by regulating the downstream target IFIT2. Interestingly, METTL3 was recently reported to regulate IFIT2 expression in a m^6^A-YTHDF2-dependent manner in cholangiocarcinoma (Xu et al., 2022). However, the difference is that their findings were in bile duct cancer. Because of the clear differences between the pathogenesis of ESCC and ICC, the distribution of m^6^A modifications in various tissue types varies widely. Whether it has the same effect is still controversial.

IFIT is a family of tetrapeptide repeat genes induced by interferon. It is commonly studied for its antiviral properties, known as an interferon-stimulated gene, and is located on the human chromosome (Pidugu et al., 2019). IFIT2 can be induced by viral infection, interferon or other pathogen-associated molecular pattern recognition and is involved in inhibiting viral replication and governing apoptosis and antitumor activity (Liu et al., 2011). IFIT2 may be crucial in controlling the inflammatory tumor environment during metastasis in OSCC, which results in cachexia (Lai et al., 2022). Koh et al. found that low IFIT2 expression in triple-negative breast cancer patients increases the risk of recurrence (Koh et al., 2019b). In particular, overexpression of IFIT2 promotes tumor cell death (Mbofung et al., 2017). IFIT2 balances pro- and antiapoptotic Bcl-2 family proteins to alter mitochondrial membrane permeability and cause apoptosis (Tait and Green, 2010; Stawowczyk et al., 2011). In colorectal cancer, IFIT2 expression is induced by IRF1, and Wnt/β-catenin signaling, which has antiapoptotic properties, inhibits it (Ohsugi et al., 2019).

In our study, a significant amount of METTL3 was detected in tissues from esophageal cancer patients compared to adjacent tissues. The METTL3 downstream target gene IFIT2 was identified in esophageal cancer cells using MeRIP-seq. *In vitro* rescue assays showed that IFIT2 could rescue the biological function of METTL3 elimination. *In vivo* research on mouse tumors using immunohistochemistry revealed a negative correlation between METTL3 and IFIT2. Furthermore, from the tail vein, we injected 1 × 10^6^ sh-METTL3 cells. The sh-NC group had liver metastases after two months (Supplementary Figure S5). Mouse lung metastases did not appear as obvious nodules (Supplementary Figure S6); this is likely because KYSE510 is a moderately aggressive cell type. When the calibration curve and ROC curve were examined, we also created a prognostic risk model using METTL3 and IFIT2 that performed well at predicting prognosis. Additionally, the immune response during the development of esophageal cancer may be mediated by METTL3 and IFIT2, which would explain why patients with esophageal cancer have a poor prognosis. These findings offer a solid foundation for future immunotherapy.

## Conclusion

In conclusion, IFIT2 and METTL3 may serve as targets for immunotherapy in addition to being potential pathogenic factors in esophageal cancer development. METTL3 and IFIT2, which may serve as prognostic or diagnostic indicators for esophageal cancer, also offer fresh evidence in favor of immunotherapy and customized treatment for ESCC patients. To assess the therapeutic potential of METTL3-regulated IFIT2 more fully in ESCC, a more thorough study with a larger sample size and multicenter clinical study should be conducted.

## Data Availability

The data presented in the study are deposited in the SRA database, accession number PRJNA889200.
