# Supplementary material for: METTL3/m6A/IFIT2 regulates proliferation, invasion and immunity in esophageal squamous cell carcinoma
Source: Front Pharmacol. 2022 Oct 20;13:1002565. doi: 10.3389/fphar.2022.1002565 (PMC9644211; doi:10.3389/fphar.2022.1002565)
Supplement: Supplementary file 1 [file Table2.DOCX]

**Supplementary** **Information for**

**METTL3/m6A/IFIT2 regulates proliferation, invasion and immunity in esophageal squamous cell carcinoma**

**Supplementary Table S1.**

**The clinical and pathological information of ESCC patients**

| \| Case \| ID \| Gender \| Age \| Grade \| Tumor type \| \| --- \| --- \| --- \| --- \| --- \| --- \| \| NO.1 \| 23193931 \| Male \| 76 \| Ⅱ-Ⅲ \| esophageal squamous cell carcinoma \| \| NO.2 \| 23191199 \| Male \| 70 \| Ⅲ \| esophageal squamous cell carcinoma \| \| NO.3 \| 23188477 \| Male \| 78 \| Ⅱ \| esophageal squamous cell carcinoma \| \| NO.4 \| 23186478 \| Male \| 80 \| Ⅰ-Ⅱ \| esophageal squamous cell carcinoma \| \| NO.5 \| 23185216 \| Male \| 73 \| Ⅱ \| esophageal squamous cell carcinoma \| \| NO.6 \| 23182588 \| Male \| 56 \| Ⅱ \| invasive squamous cell carcinoma of  esophagussus \| \| NO.7 \| 23177655 \| Male \| 54 \| Ⅱ-Ⅲ \| esophageal squamous cell carcinoma \| \| NO.8 \| 11960383 \| Male \| 52 \| Ⅱ \| esophageal squamous cell carcinoma \| \| NO.9 \| 11974562 \| Male \| 75 \| Ⅱ-Ⅲ \| esophageal squamous cell carcinoma \| \| NO.10 \| 11975688 \| Male \| 58 \| Ⅱ-Ⅲ \| esophageal squamous cell carcinoma \| \| NO.11 \| 11704386 \| Famale \| 73 \| Ⅱ-Ⅲ \| esophageal squamous cell carcinoma \| |  |  |  |  |  |
| --- | --- | --- | --- | --- | --- | --- | --- | --- | --- | --- | --- | --- | --- | --- | --- | --- | --- | --- | --- | --- | --- | --- | --- | --- | --- | --- | --- | --- | --- | --- | --- | --- | --- | --- | --- | --- | --- | --- | --- | --- | --- | --- | --- | --- | --- | --- | --- | --- | --- | --- | --- | --- | --- | --- | --- | --- | --- | --- | --- | --- | --- | --- | --- | --- | --- | --- | --- | --- | --- | --- | --- | --- | --- | --- | --- | --- | --- |

**Supplementary Table S2**

**Information of sequences used in this study**

| **Group** | **Names** | **Sequences** |
| --- | --- | --- |
| q-RT PCR primer | h-METTL3-F | 5'-TGTGATCGTAGCTGAGGTTCGT-3' |
|  | h-METTL3-R | 5'-TGATCCAGTTGGGTTGCACATT-3' |
|  | h-METTL3-Probe | 5'-TCCAAA+TAAC+TCAA+TC+TTGCGAG+TGCC-3' |
|  | h-IFIT2-F | 5'-TACATACCAAACAATGCCTACCTG-3' |
|  | h-IFIT2-R | 5'-GAGCCACAGCGTGTCCTATTAG-3' |
|  | h-IFIT2-Probe | 5'-TGCTGCTATAGGGCAAAAGTCTTCCAAG-3' |
|  | h-ACTB-F | 5'-GCCCATCTAXGAGGGGTATG-3' |
|  | h-ACTB-R | 5'-GAGGTAGTCAGTCAGGTCCCG-3' |
|  | h-ACTB-Probe | 5'-CY5-CCCCCATGCCATCCTGCGTC-3' |
| Knockdown METTL3 Sequences | Negative Control-F | 5'-GATCCGTTCTCCGAACGTGTCACGTAATTCAAGAGATTACGTGACACGTTCGGAGAATTTTTTC-3' |
|  | Negative Control-R | 5'-AATTGAAAAAATTCTCCGAACGTGTCACGTAATCTCTTGAATTACGTGACACGTTCGGAGAACG-3' |
|  | sh-METTL3-1-F | 5'-GATCCGGAGGAGTGCATGAAAGCCAGTGATTTCAAGAGAATCACT GGCTTTCATGCACTCCTCCTTTTTTG-3' |
|  | sh-METTL3-1-R | 5'-AATTCAAAAAAGGAGGAGTGCATGAAAGCCAGTGATTCTCTTGAA ATCACTGGCTTTCATGCACTCCTCCG-3' |
|  | sh-METTL3-2-F | 5'-GATCCGCCCACCTCAGTGGATCTGTTGTGATTTCAAGAGAATCACA ACAGATCCACTGAGGTGGGTTTTTTG-3' |
|  | sh-METTL3-2-R | 5'-AATTCAAAAAACCCACCTCAGTGGATCTGTTGTGATTCTCTTGAAA TCACAACAGATCCACTGAGGTGGGCG-3' |
|  | sh-METTL3-3-F | 5'-GATCCGCCCACCCTGGGATATTCACATGGAATTCAAGAGATTCCAT GTGAATATCCCAGGGTGGGTTTTTTG-3' |
|  | sh-METTL3-3-R | 5'-AATTCAAAAAACCCACCCTGGGATATTCACATGGAATCTCTTGAAT TCCATGTGAATATCCCAGGGTGGGCG-3' |
| Knockdown IFIT2 Sequences | Negative control-F | 5'- UUCUCCGAACGUGUCACGUTT-3' |
|  | Negative control-R | 5'-ACGUGACACGUUCGGAGAATT-3' |
|  | si-IFIT2-1-F | 5'-GCCAGACAAAGCGAUUGAATT-3' |
|  | si-IFIT2-1-R | 5'-UUCAAUCGCUUUGUCUGGCTT-3' |
|  | si-IFIT2-2-F | 5'-CUCAGACGUUCAGAUUUAUTT-3' |
|  | si-IFIT2-2-R | 5'-AUAAAUCUGAACGUCUGAGTT-3' |
|  | si-IFIT2-3-F | 5'-CCUACCUGCAUUGCCAAAUTT-3' |
|  | si-IFIT2-3-R | 5'-AUUUGGCAAUGCAGGUAGGTT-3' |

# Supplementary Figures:

Figure S1


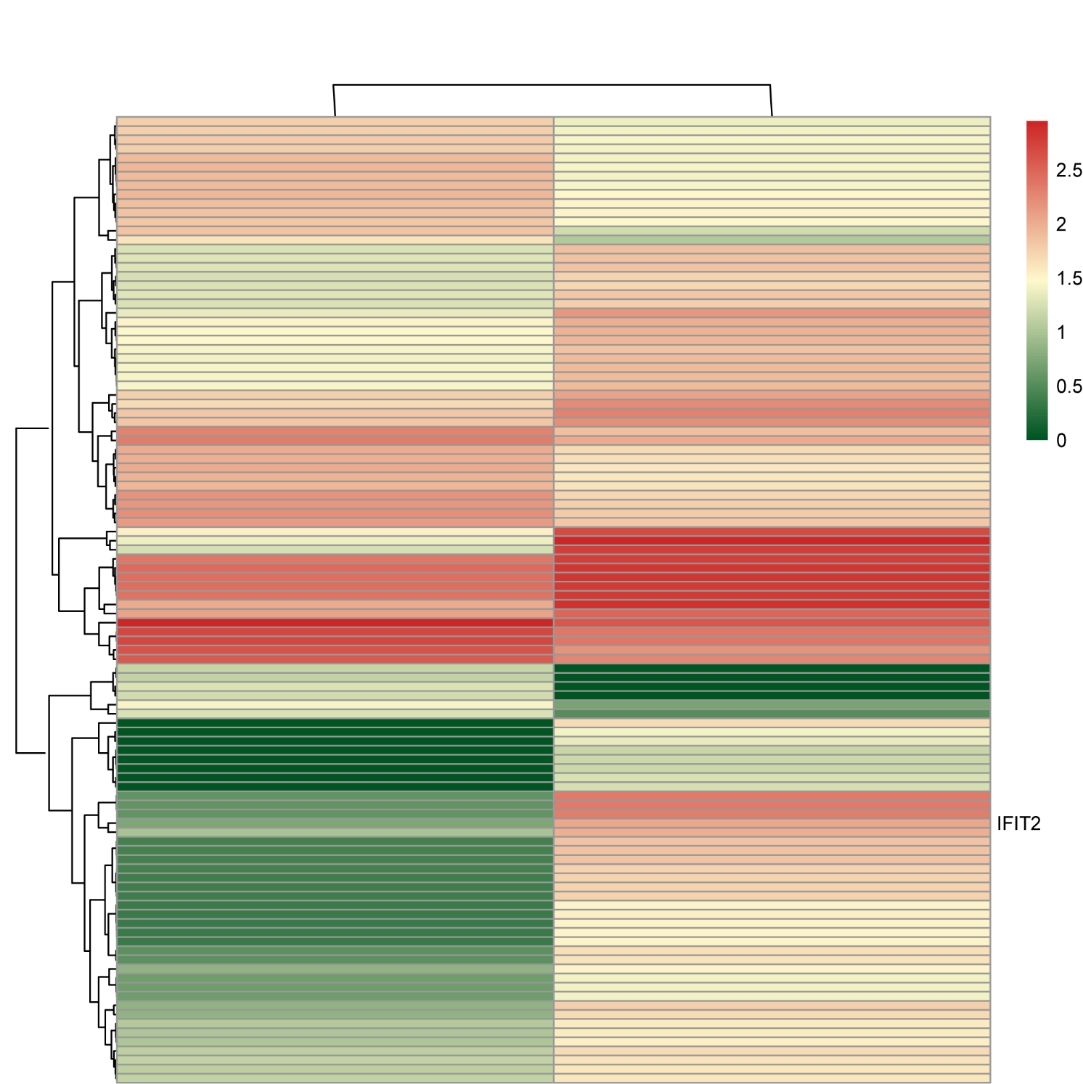


Figure S2


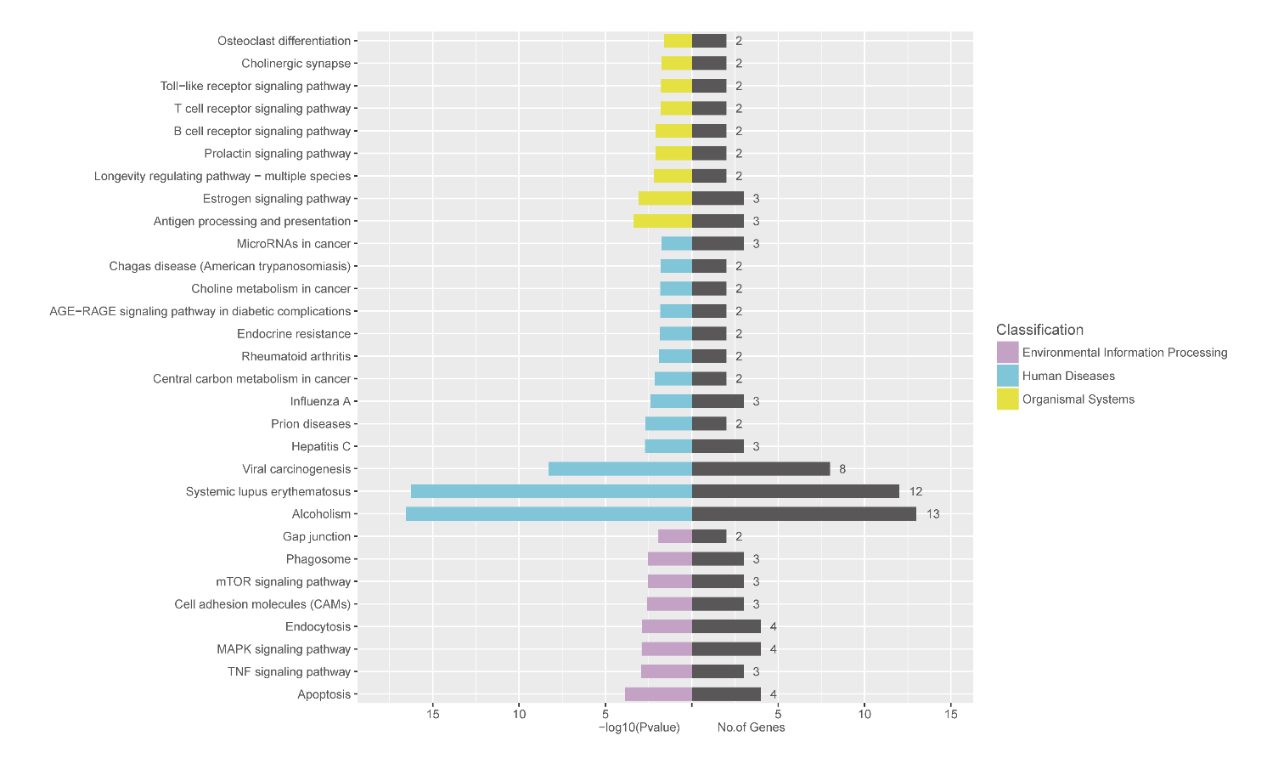


Figure S3


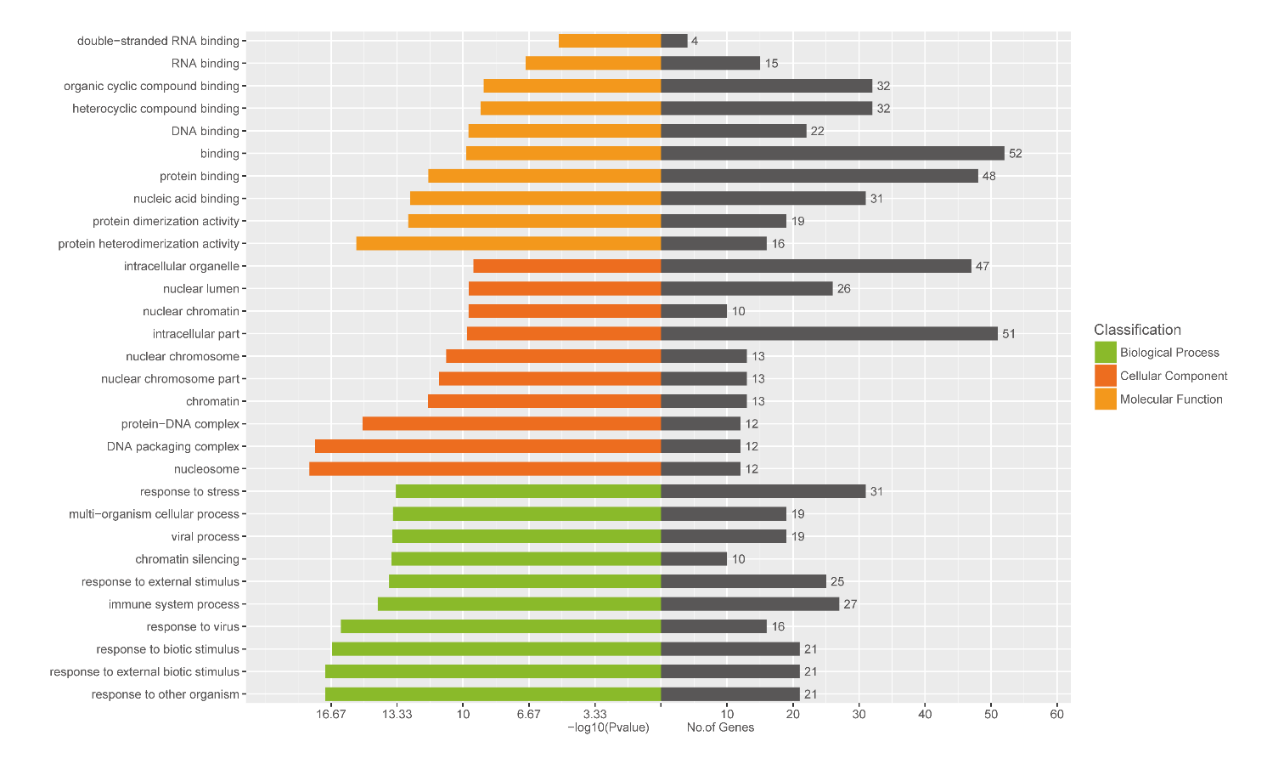


Figure S4


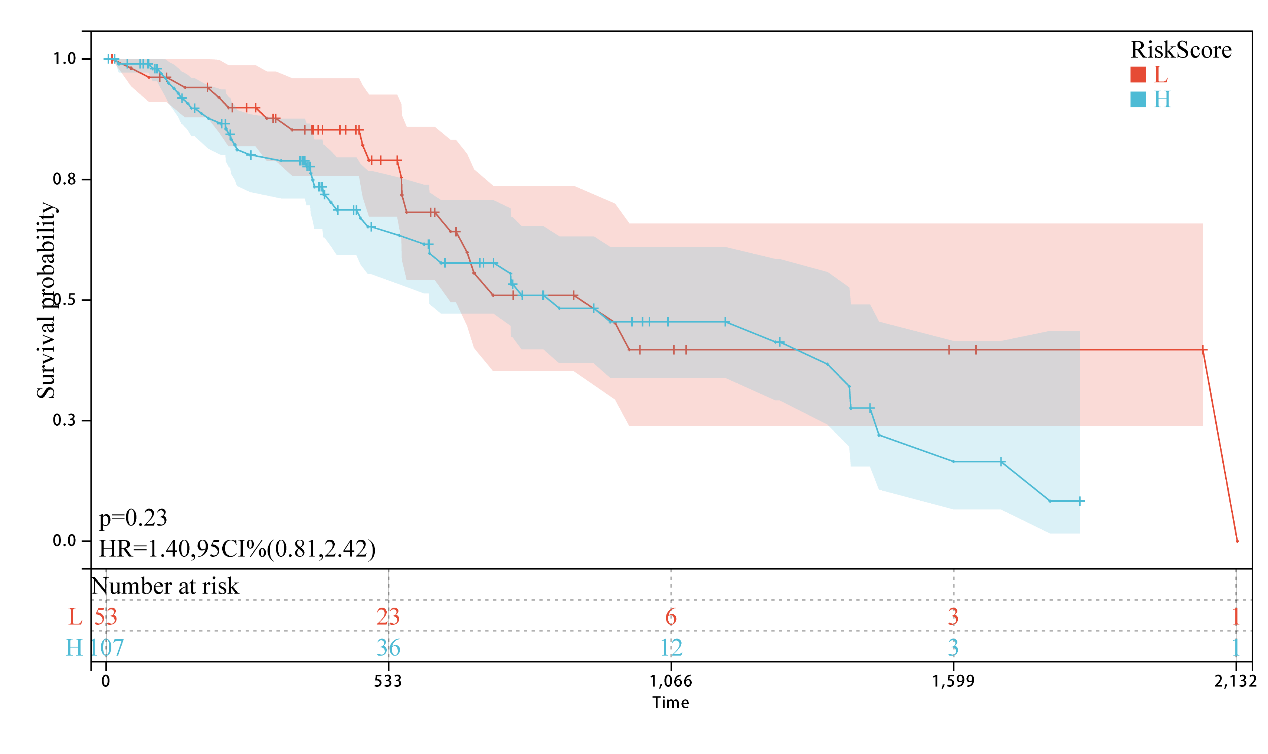


Figure S5


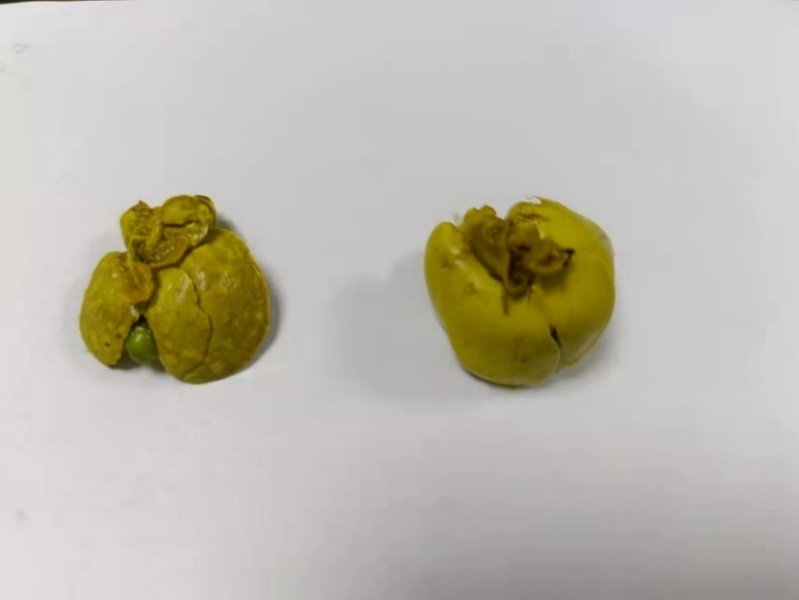


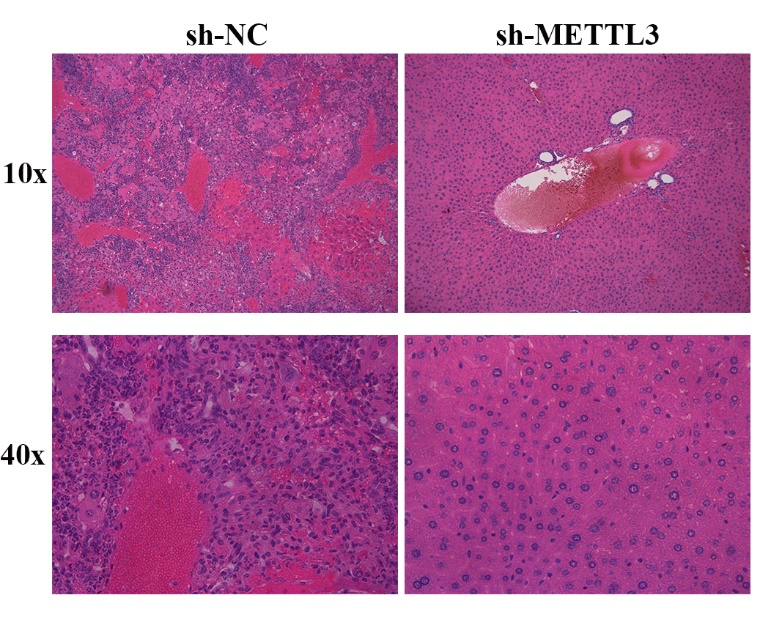


**Hepatic nodules in sh-NC group .**

Figure S6


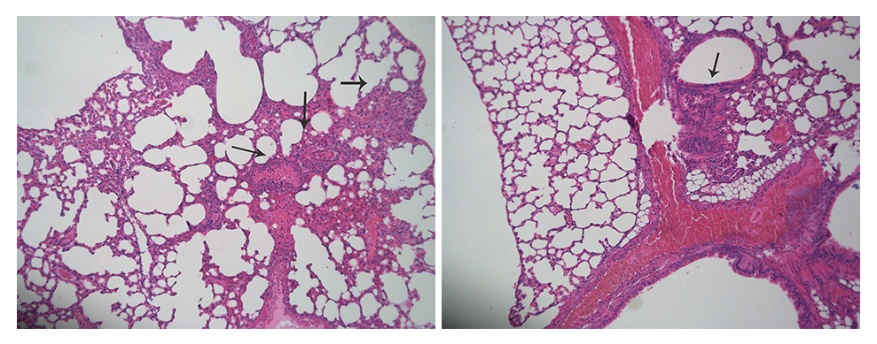


**Pulmonary nodule indicated by arrow.**

Figure S7


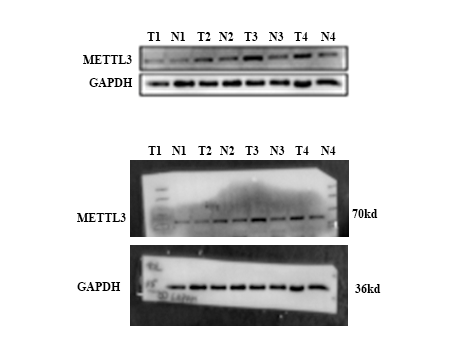
 **
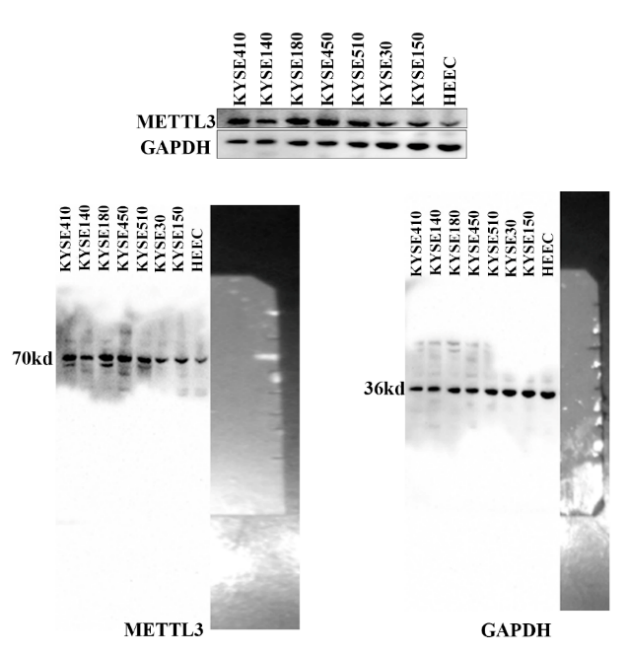
**

**METTL3 in Figure 1B,D**.

Figure S8

**
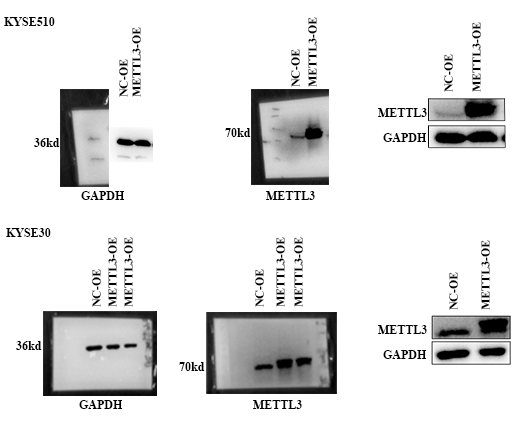
**

Figure S9


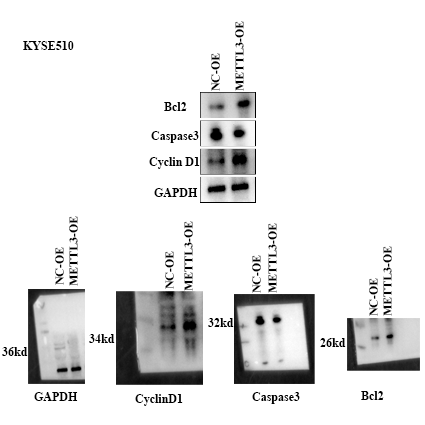
 **
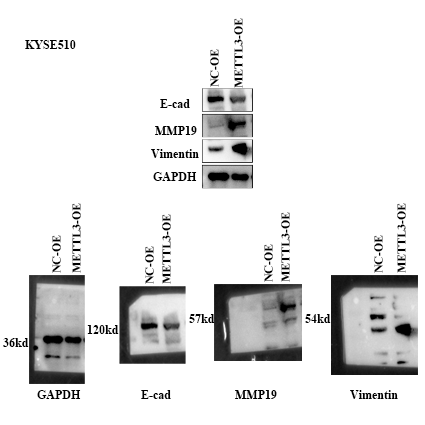
**

**METTL3 in METTL3-OE cell and Bcl2,Caspase3,CyclinD1,E-cad,MMP19,Vimentin in Figure 2H.**

Figure S10

**
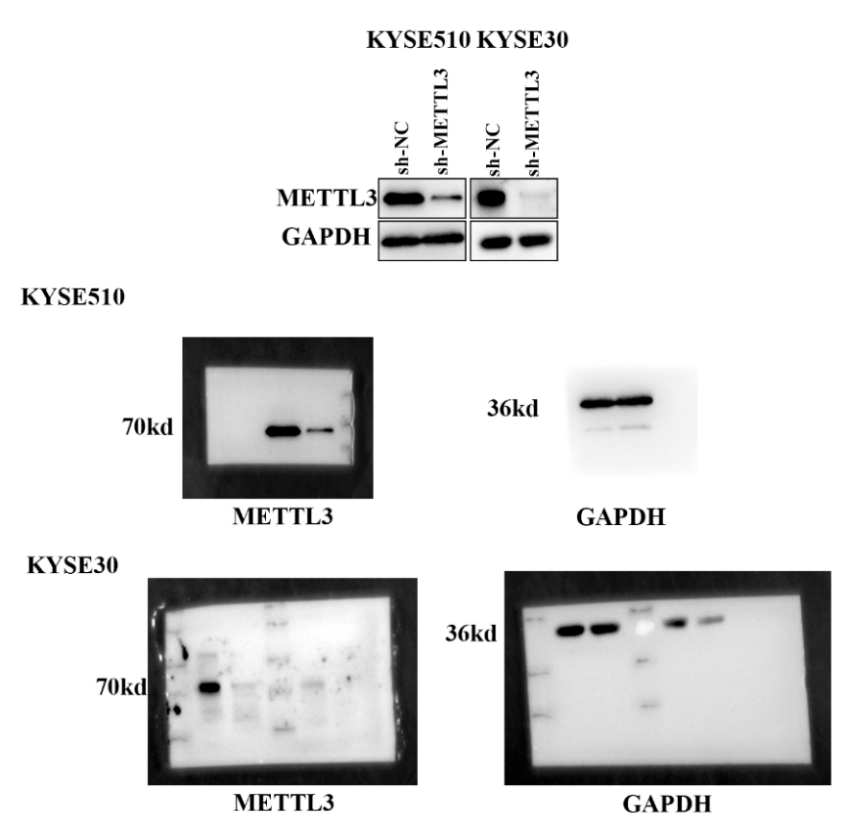
**

**
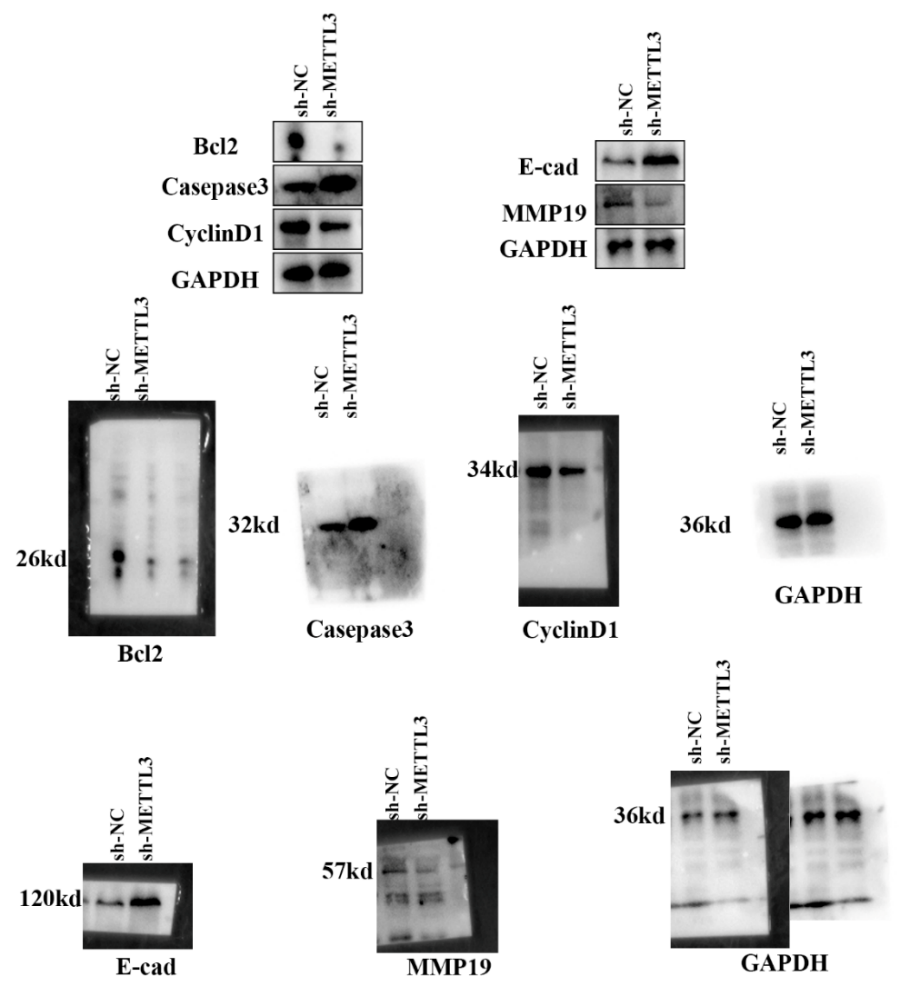
**

**METTL3 in sh-METTL3 cell and Bcl2,Caspase3,CyclinD1,E-cad,MMP19,Vimentin in sh-METTL3 cell.**

Figure S11

**
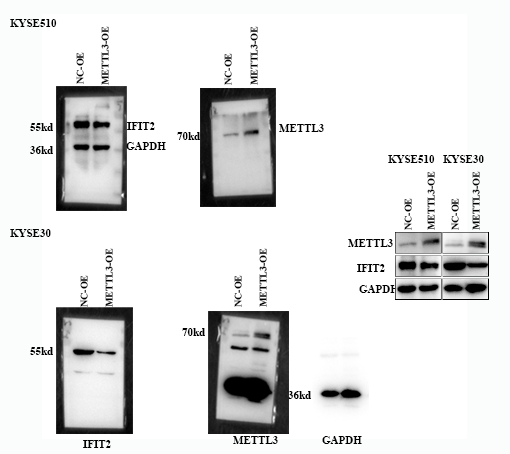
**

**IFIT2 in METTL3-OE cells in Figure 4I**

**
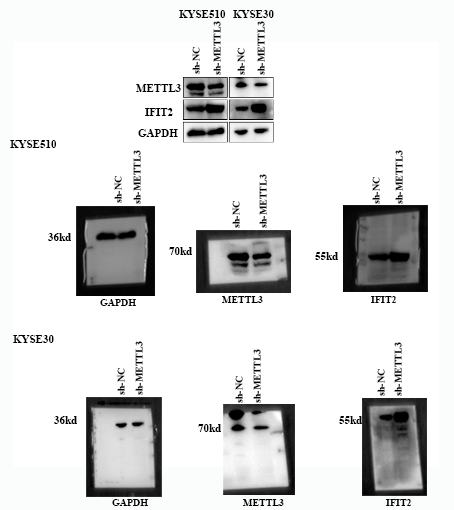
**

**IFIT2 in sh-METTL3 cells in Figure 4I.**

Figure S12


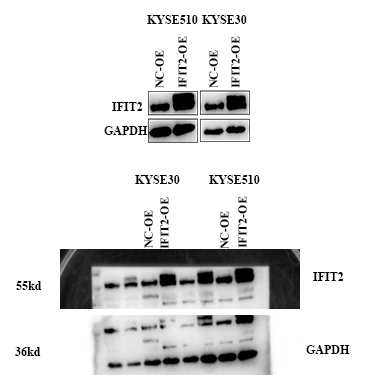


Figure S13

**IFIT2 in Figure 5B**.


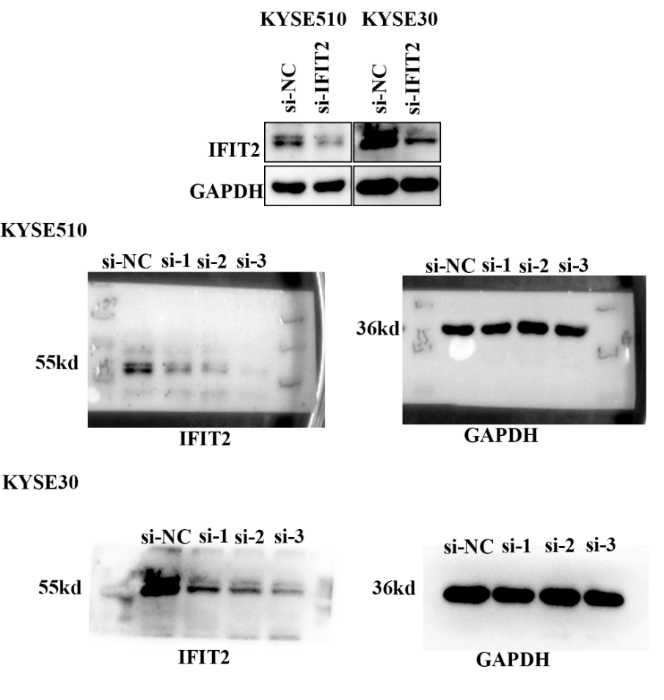


**IFIT2 in Figure 6B**.

Figure S14

**
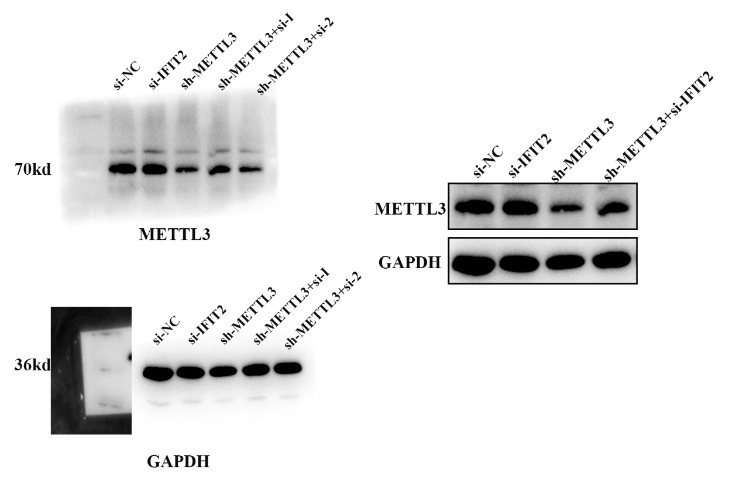
**

**IFIT2 in Figure 7A**.

Figure S15

**
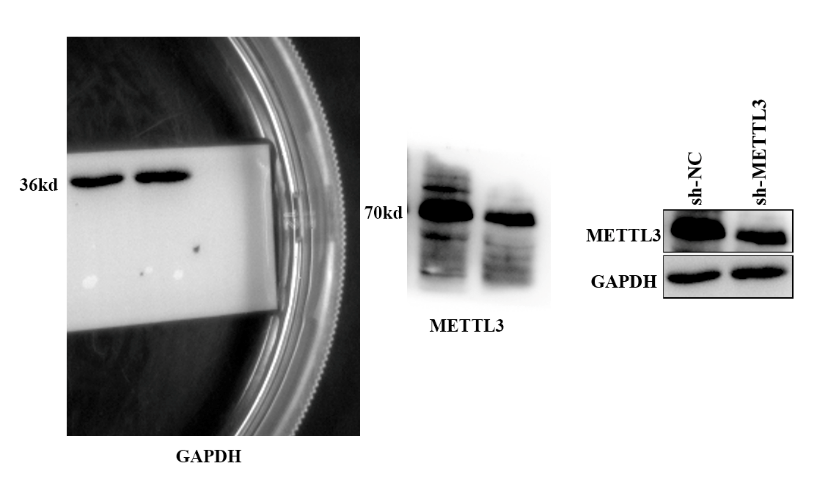
**

**METTL3 in mice Figure 8D.**
